# Mapping Ligand Interactions of Bromodomains BRD4 and ATAD2 with FragLites and PepLites—Halogenated Probes of Druglike and Peptide-like Molecular Interactions

**DOI:** 10.1021/acs.jmedchem.2c01357

**Published:** 2022-11-11

**Authors:** Gemma Davison, Mathew P. Martin, Shannon Turberville, Selma Dormen, Richard Heath, Amy B. Heptinstall, Marie Lawson, Duncan C. Miller, Yi Min Ng, James N. Sanderson, Ian Hope, Daniel J. Wood, Céline Cano, Jane A. Endicott, Ian R. Hardcastle, Martin E. M. Noble, Michael J. Waring

**Affiliations:** † Cancer Research Horizons Therapeutic Innovation, Newcastle Drug Discovery Unit, Newcastle University Centre for Cancer, Chemistry, School of Natural and Environmental Sciences, Newcastle University, Bedson Building, Newcastle upon Tyne NE1 7RU, U.K.; ‡ Cancer Research Horizons Therapeutic Innovation, Newcastle Drug Discovery Unit, Newcastle University Centre for Cancer, Newcastle University, Paul O’Gorman Building, Framlington Place, Newcastle upon Tyne NE2 4AD, U.K.

## Abstract

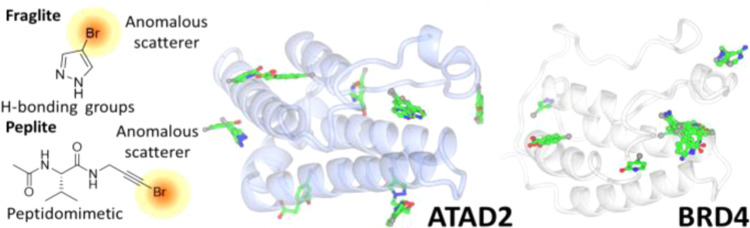

The development of ligands for biological targets is critically dependent on the identification of sites on proteins that bind molecules with high affinity. A set of compounds, called FragLites, can identify such sites, along with the interactions required to gain affinity, by X-ray crystallography. We demonstrate the utility of FragLites in mapping the binding sites of bromodomain proteins BRD4 and ATAD2 and demonstrate that FragLite mapping is comparable to a full fragment screen in identifying ligand binding sites and key interactions. We extend the FragLite set with analogous compounds derived from amino acids (termed PepLites) that mimic the interactions of peptides. The output of the FragLite maps is shown to enable the development of ligands with leadlike potency. This work establishes the use of FragLite and PepLite screening at an early stage in ligand discovery allowing the rapid assessment of tractability of protein targets and informing downstream hit-finding.

## Introduction

**Figure 1:**
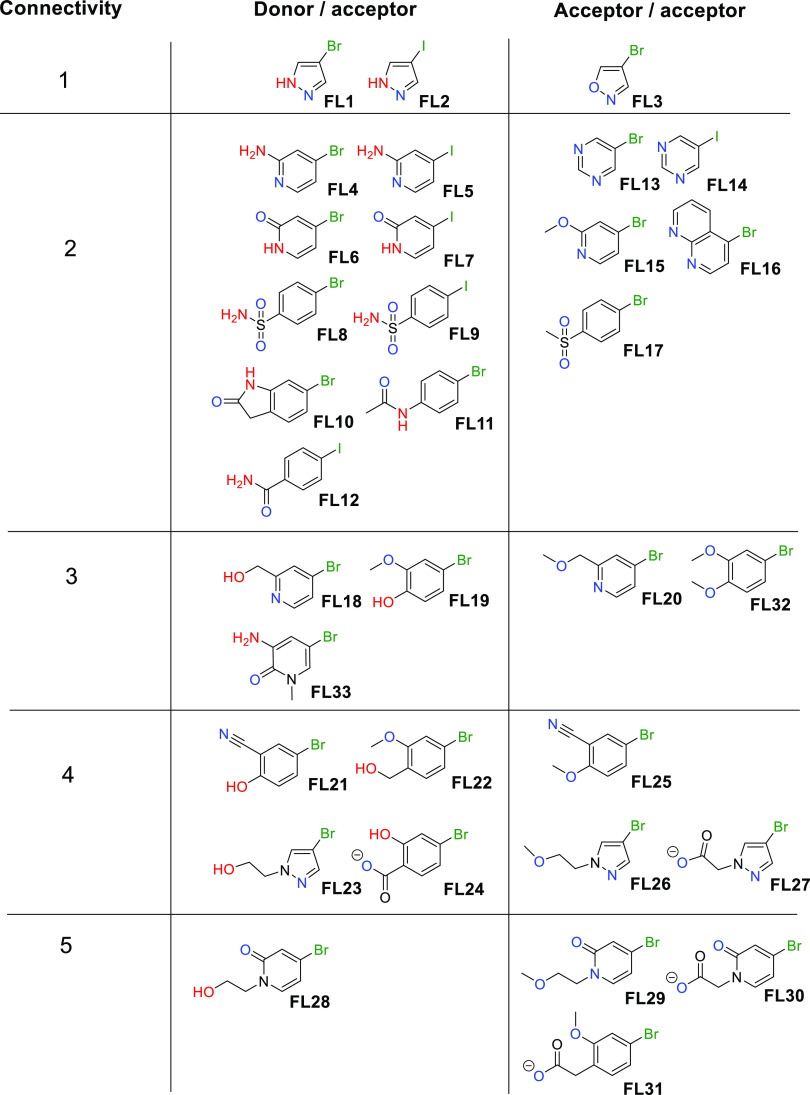
FragLite compound set aligned by hydrogen-bond donor/acceptor array.

Structural characterization of the interactions between proteins and their natural or unnatural ligands is vital for the elucidation of their biological function and their exploitation as therapeutic targets.^1^ Perhaps the most effective way of establishing sites of ligand interaction is by compound screening and subsequent structural biology.^2,3^ In some cases, this requires extensive testing of druglike or fragment-like libraries, which is resource-intensive due to the number of compounds that need to be screened to cover a sufficient range of chemical diversity.^4^ To provide a faster, less resource-intensive means of mapping interaction sites, we have recently described the use of FragLites, a small set of very simple compounds displaying hydrogen-bonding motifs, specifically proximal combinations of two hydrogen-bonding groups, designed to permit the co-operative formation of hydrogen bonds.^5^ Similar approaches using minimal fragments have been described by others.^6^ Critically, all FragLites incorporate a heavy halogen atom (Figure 1)^5^ that enables the characterization of ligand-bound X-ray structures unambiguously by observation of anomalous dispersion.^7^ Initially, the FragLite concept was illustrated with mapping of CDK2, with a focus on investigating interactions of druglike small molecules. To further develop this approach, we wished to explore a wider range of proteins.

**Figure 2:**
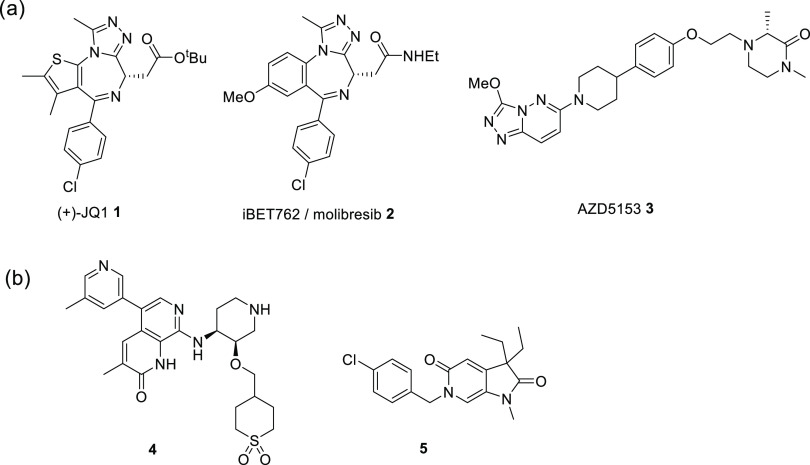
Literature inhibitors of (a) BRD4 and (b) ATAD2.

Bromodomain-containing proteins are a prominent class of epigenetic readers that recognize acetylated lysine residues, typically on histones.^1^ BRD4, a member of the bromodomain and extraterminal subfamily, which in fact contains two bromodomains, is perhaps the most widely studied family member and has been shown to be very amenable to binding druglike molecules. A number of BRD4 inhibitors have been discovered, such as the chemical probe (+)-JQ1 **1**(8) and several, including molibresib **2**(9) and AZD5153 **3**,^10,11^ have progressed into clinical trials (Figure 2a). ATAD2 has also been the subject of the development of chemical probes and drug discovery (Figure 2b).^12,13^ ATAD2 was proposed from computational druggability analysis to be less tractable than BRD4, attributed in part to it lacking the hydrophobic region present in the BRD4 ligand binding site, termed the “WPF shelf”, interactions with which are postulated to contribute significantly to ligand binding affinity.^14^ This initial view is reflected in subsequent ligand development work in which it has proven harder to find high-affinity ligands for ATAD2 than for BRD4. Nevertheless, potent ligands for ATAD2, such as **4**,^12^**5**,^15^ and others,^16^ have been found.

In part due to this difference in “ligandability”, we selected BRD4 and ATAD2 as a pair of proteins to demonstrate the potential of FragLite mapping. The ability of FragLites to identify the ligand binding site for both proteins would further validate their use in identifying tractable binding sites in novel proteins. Moreover, the observation of greater numbers of hits for BRD4 compared to ATAD2 would show, for the first time, that FragLites can be used to quantify relative ligandability between different proteins.

## Results

### Mapping of BRD4 and ATAD2

**Figure 3:**
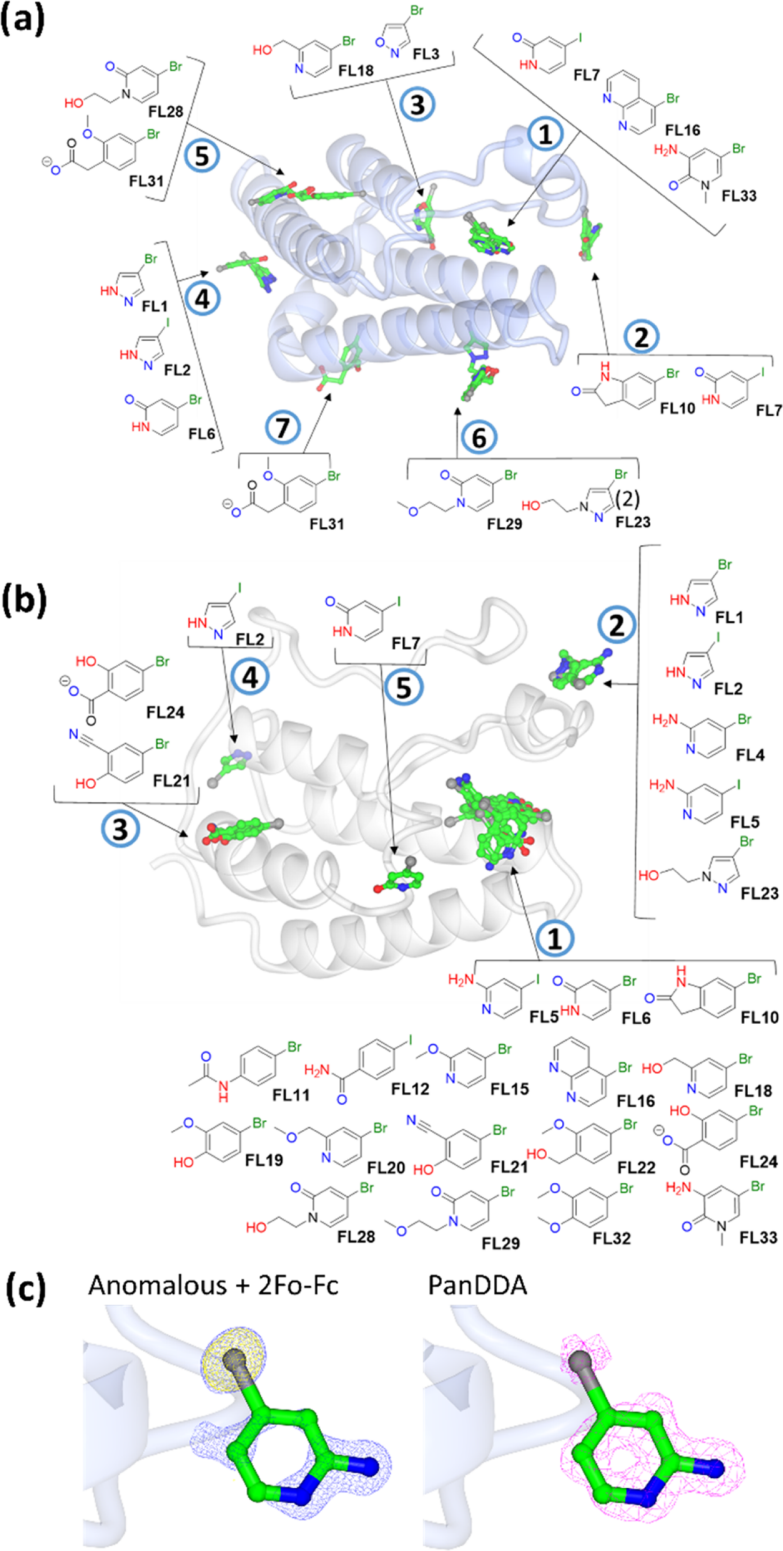
FragLite screening of (a) ATAD2 (PDB: 7QUK, 7QUM, 7PPX, 7QWO, 7QX1, 7QXT, 7QU7, 7QYK, 7QYL, 7QZM,7QZY, 7QZZ, 7R00) and (b) BRD4 (PDB: 7Z9W, 7Z9Y, 7ZA6, 7ZA7, 7ZA8, 7ZA9, 7ZE6, 7ZAA, 7ZAD, 7ZAE, 7ZAJ, 7ZAR, 7ZAQ, 7ZAT, ZE7, 7ZEF, 7ZEN, 7ZFN, 7ZFO, 7ZFQ, 7ZFS, 7ZFT). (c) Anomalous, PanDDA, and 2Fo-Fc maps of **FL4** bound to BRD4 site 2. Site 1 represents the orthosteric KAc binding site.

Members of the FragLite library (Figure 1) were soaked individually into ATAD2 and BRD4 (first bromodomain), and the resulting crystals were analyzed by X-ray diffraction. Inspection of anomalous peaks greater than 5 standard deviations above the mean led to the identification of 6 binding events across 5 sites for ATAD2 and 21 binding events across 5 sites for BRD4 (Figure 3).

To assess the sensitivity of FragLite detection, we sought to compare the hits found from inspection of anomalous maps with those found by the PanDDA algorithm, a statistical approach that identifies and deconvolutes binding events in normal (i.e., not anomalous) electron density maps.^17^ Accordingly, we compared our initial 5-sigma anomalous hits with those identified in a default run of PanDDA. In addition to those sites found from the anomalous map inspection, PanDDA was able to identify a further 10 binding events for ATAD2 and identified an additional 3 sites, and a further 5 binding events for BRD4 across the previously identified sites (Table S1). Investigating the anomalous maps contoured around the location of FragLites found by PanDDA revealed that anomalous peaks greater than 3 standard deviations above the mean were located at the corresponding halogen atomic positions. This suggests that a threshold of 3 standard deviations might be more suitable for screening maps for hits, although some false positives would be expected if this less stringent criterion is applied.

Interestingly, the quality and quantity of information derived from FragLite mapping are maximized when both PanDDA and anomalous signals are combined. As an illustration, PanDDA identified a binding event for **FL1** that was not recognized from the inspection of anomalous maps and did not define an unambiguous bound pose in the PanDDA event map (Figure 3c). Only when information from the two approaches was used in combination could a clear binding event be observed.

The overall binding event counts of 16 across 7 sites for ATAD2, and 26 across 5 sites for BRD4 (Figure 3), represent a relatively high hit rate on domains of ca. 20 kDa, consistent with the count of 9 binding events across 6 sites previously observed for FragLite mapping of CDK2.

### FragLite Interactions

**Figure 4:**
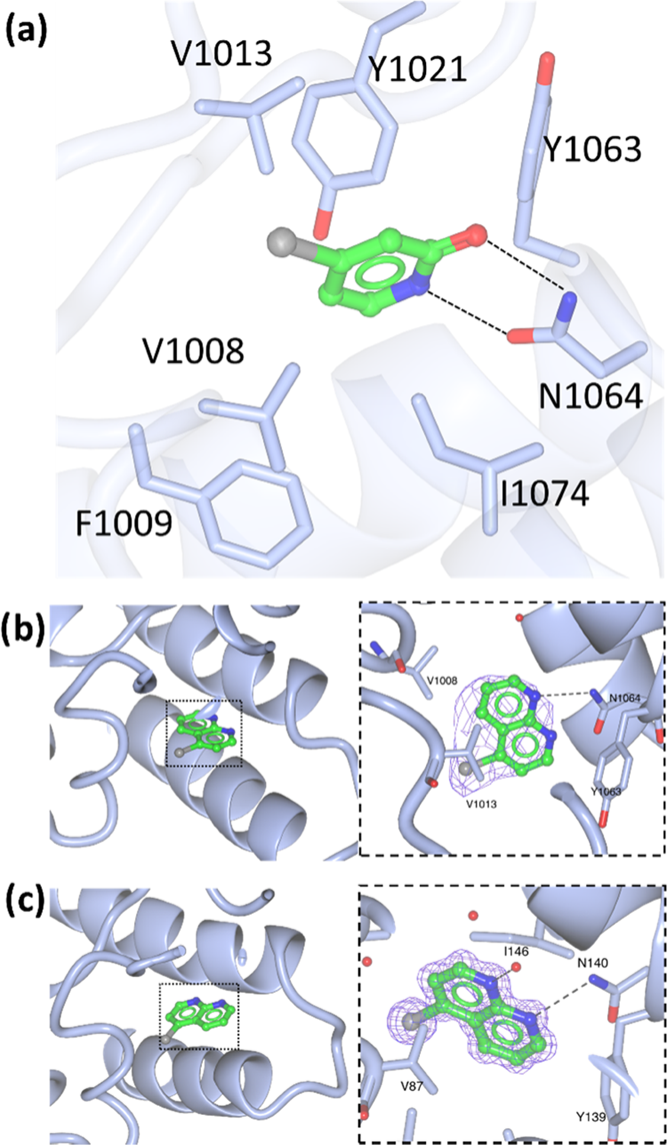
(a) **FL7** bound to the ATAD2 KAc pocket (PDB: 7QX1); (b)Interaction of **FL16** with BRD4 site 1 (PDB: 7ZAJ); (c) **FL16** interactions with ATAD2 site 1 (PDB: 7QU7).

Systematic analysis of hydrogen bonding in FragLites that target ATAD2 and BRD4 confirms that FragLites generally form one or more hydrogen bonds (average 1.1 per bound pose). Critical interactions made by bromodomain ligands were identified by the FragLites in the orthosteric site. Most significantly, hydrogen bonds to the asparagine residue in the orthosteric site (Asn1064, ATAD2/Asn140, BRD4) were identified by multiple FragLites. For example, bifurcated hydrogen bonds akin to those made by **4** were formed in an analogous way with **FL7** (Figure 4a). Dual acceptor FragLite **FL16** formed single H-bond interactions with the Asn-NH_2_ in both proteins (Figure 4b,c). In addition to their hydrogen bonding interactions, all of the FragLite hits exploit lipophilic interactions of their heterocyclic core (e.g., **FL7**, Figure 4a).

Although the intended role of the bromine or iodine atom in FragLites is to aid with the identification of the location and orientation of the hits, and ideally will not be involved in the binding directly, halogen interactions are also observed in the binding modes of authentic drugs and chemical probes.^18^ Consistent with this role, the halogen atoms of FragLites are seen to form contacts with the protein in 33 of the 42 binding events. However, a halogen bond constitutes the primary interaction (other than lipophilic interactions) in the absence of a hydrogen bond only for **FL10** and **23** in ATAD2, and **FL2**, **4**, **21**, and **24** for BRD4 (Table S1).

The relevance of FragLite mapping for characterizing the druggability of a target, identifying interaction hotspots, or providing leads for the development of drugs and chemical probes, depends on the hits being reflective of interactions that might occur in solution as well as in protein crystals. In both ATAD2 and BRD4, while the majority (22/42) of binding events involved ligands that contacted only one molecule, a significant fraction (**FL2**, **4**, **5**, **7**, **10**, **11**, **12**, **15**, **19**, **21**, **22**, and **24** in BRD4 and **FL1**, **2**, **6**, **7**, **10**, **28**, and **29** in ATAD2) were involved in contacts with more than one protein molecule in the lattice (Table S1). As such, the hits for these FragLites may not occur in solution. A benefit of the crystallographic fragment screening approach is that such possible false positives are readily identified. In assessing the potential for binding to protein in solution, such hits should likely be discarded. Assessment of their potential for binding to monomeric protein in solution may be possible via biophysical techniques, but the FragLites will often not have sufficient affinity for such experiments to be informative.

### FragLite Pockets

We next evaluated FragLite hits against BRD4 and ATAD2 to see whether they occurred preferentially in the orthosteric (i.e., acetyl lysine binding) or an allosteric site. Three FragLites (**FL7**, **16**, and **33**) were identified in the orthosteric site of ATAD2 and 17 FragLites (**FL5**, **6**, **10**, **11**, **12**, **15**, **16**, **18**, **19**, **20**, **21**, **22**, **24**, **28**, **29**, **32**, and **33**) were observed in the BRD4 orthosteric site (Figure 3). Those FragLites identified in the orthosteric site of either ATAD2 or BRD4 recapitulate a key interaction made by the endogenous and known synthetic bromodomain ligands with a conserved asparagine residue (Asn1064 in ATAD2, Asn140 in BRD4). The frequency of hits in the orthosteric sites confirms the ability of FragLites to identify the functional binding site of a protein, as hinted at by the previously described mapping of the ATP binding site of CDK2,^5^ but crucially here extended beyond identifying a co-factor binding site to identifying a site of protein–protein interaction.

The remaining hits for ATAD2 (13/16) and BRD4 (9/26) were bound in allosteric pockets of their respective proteins (Figure 3, Table S1). This confirms that the FragLite library has the potential to identify alternative small molecule-binding pockets. These pockets may correspond to surfaces that mediate protein–protein interactions that are important for the recognition of molecular partners.

### Comparison with Full Fragment Screen

**Figure 5:**
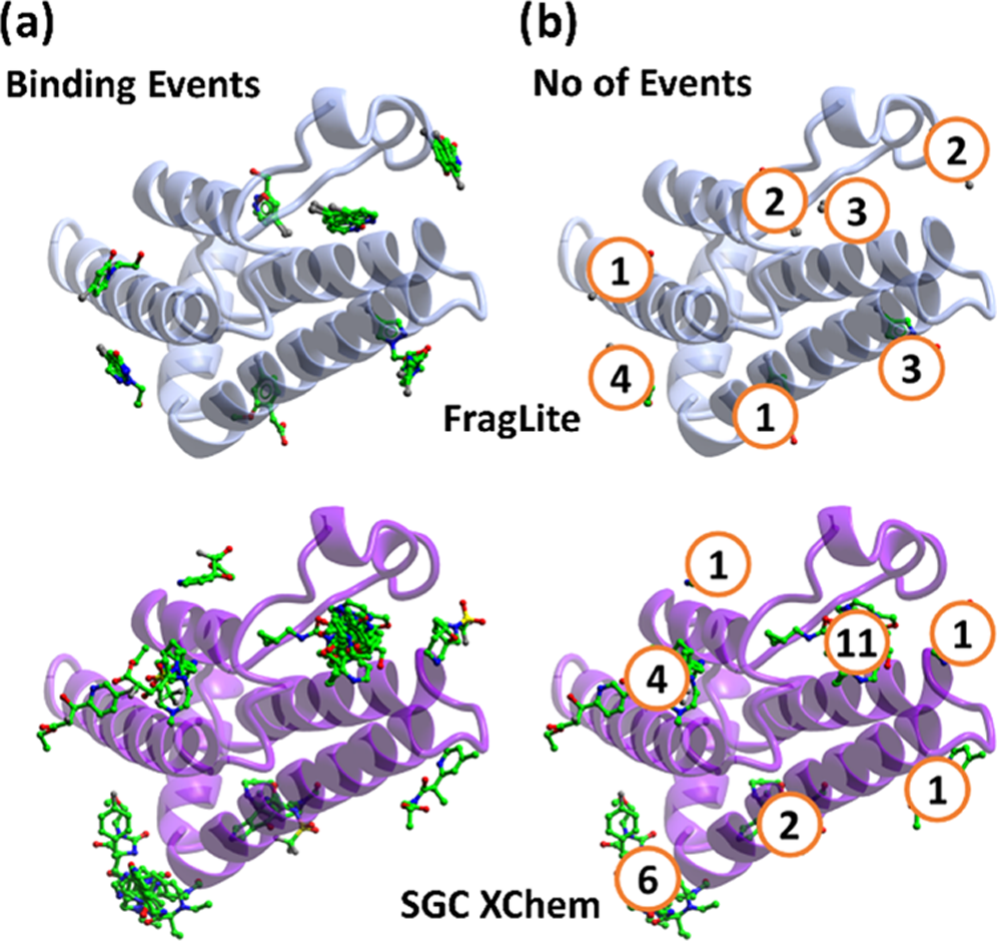
Library benchmarking against ATAD2. (a) Binding event of FragLite and SGC XChem libraries. (b) Number of binding events per site for FragLite and SGC XChem libraries.

To determine whether FragLite mapping can provide comprehensive coverage of hotspots on a target, we compared the output of a FragLite experiment with the results found with a much larger fragment library, namely, the 776 compound DSI-poised library from the Diamond Light Source XChem facility.^19^ When comparing the ATAD2 hits obtained using FragLites to those obtained with the XChem library, we found almost exact correspondence between sites (Figure 5). As expected from a large and mature fragment collection, the XChem library screening provided more hits in each site. Six sites were identified by both libraries, while each library identified one site that was not seen with the other.

### PepLites

The role of many regulatory domains, including the bromodomains explored here, is to recognize peptide motifs. Interference with these protein–peptide (or protein–protein) interactions is one route to modulating biological activity, either for therapeutic or investigative purposes. Additionally, a further use of this type of approach could be to find protein–protein interaction hotspots. Because the FragLite library is designed based on small-molecule druglike interactions, to better represent peptide-like interactions in our screening library, we determined that the screening set should be expanded to encompass molecules that recapitulate amino acid sidechain contacts. Accordingly, we designed a set of capped amino acids containing bromine tags.

**Figure 6:**
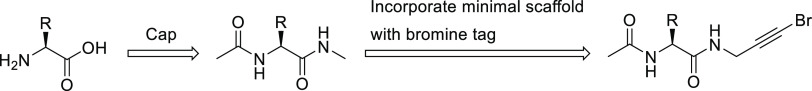
Design of PepLites.

Incorporation of an inert bromine substituent in amino acids is difficult, since on sp^3^-hybridized carbon atoms, they are prone to nucleophilic substitution and elimination reactions. We therefore incorporated the bromine atom into a 1-bromoacetylene motif appended to the C-terminal amino capping group (Figure 6) as brominated sp-carbon atoms are stable to elimination and significantly less prone to nucleophilic attack. These amino acid equivalents of the FragLites were colloquially termed PepLites.

**Figure 7:**
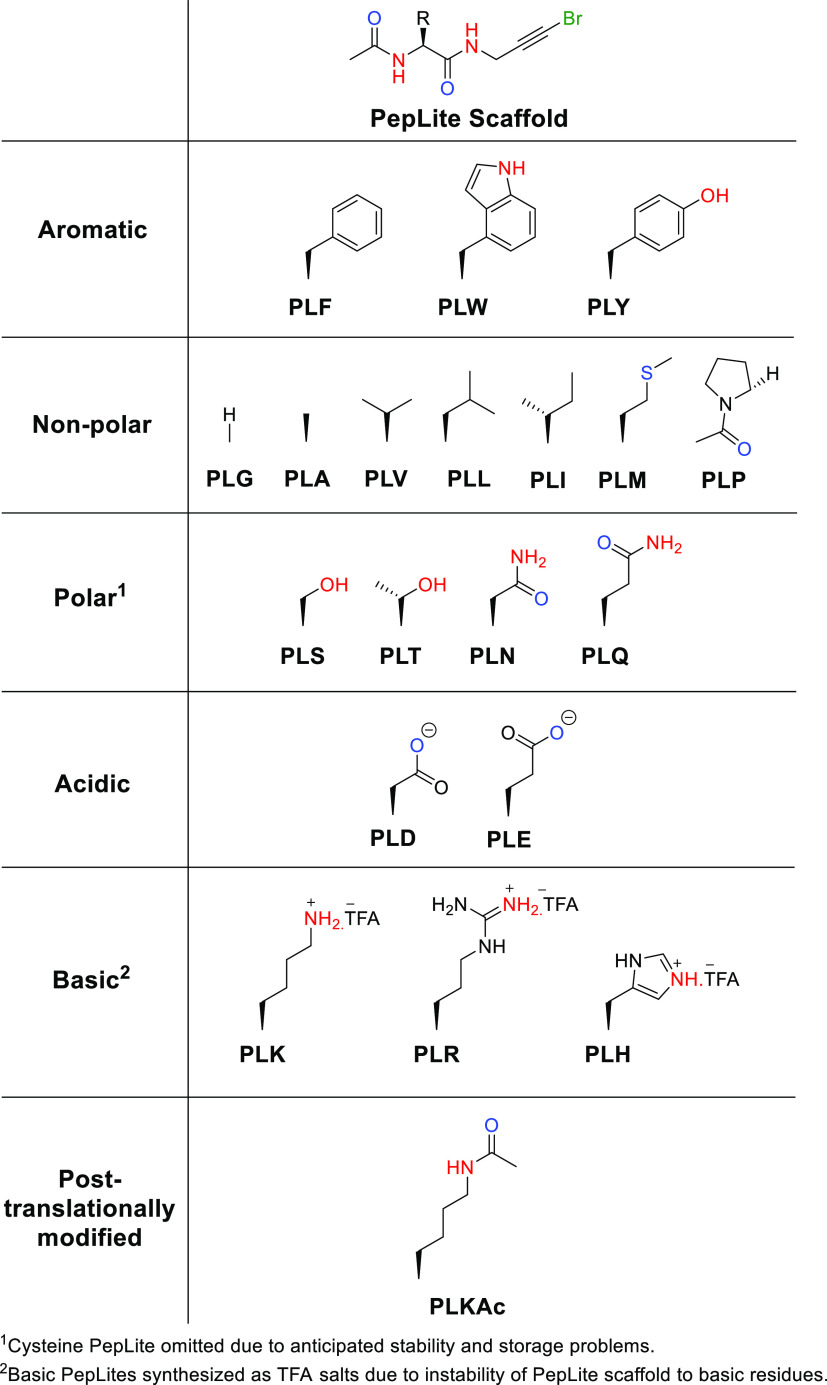
PepLite compound set. Labeled as PL followed by their amino acid single-letter code.

**Scheme 1:**
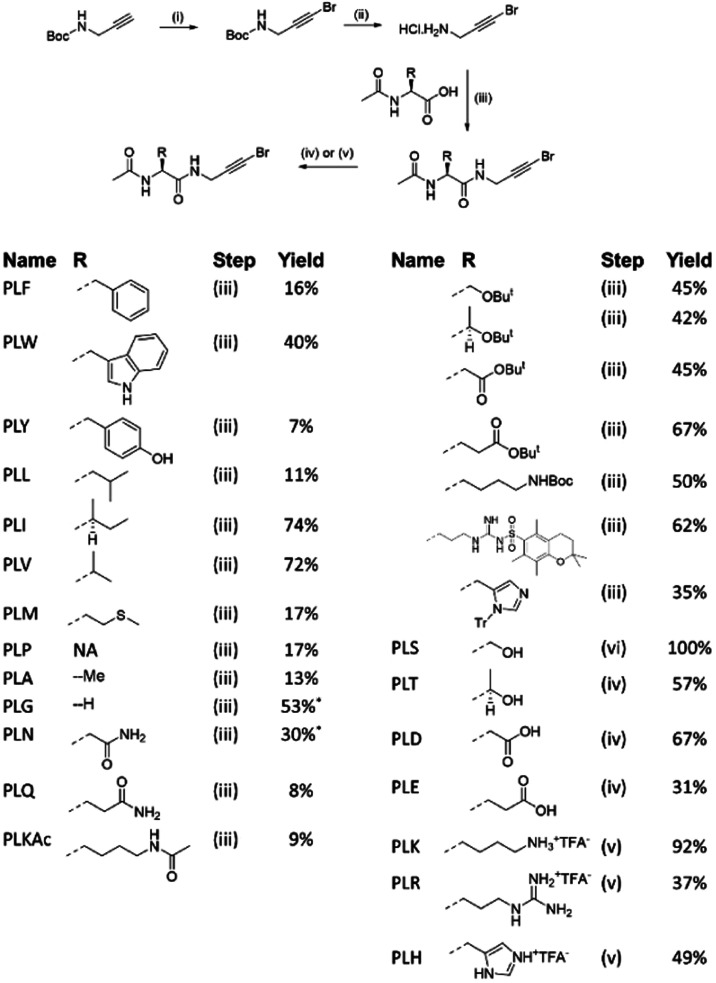
Synthesis of PepLite Library, Conditions (i) KOH, Br_2_, MeOH, 0 °C to rt, 79%; (ii) 4 M HCl in dioxane, rt, 100%; (iii) HATU, DIPEA, DMF, 40 °C; (iv) 2:1 TFA/DCM, 0 °C to rt; (v) 10:1:1 TFA/TIPSH/water, rt. Pyridine used in place of DIPEA.

A set of canonical and post-translationally modified (acetyl lysine) amino acids bearing 1-bromopropargylamide at the C-terminus and acetylated on the N-terminus were prepared (Figure 7 and Scheme 1, labeled as ***PL*** followed by their amino acid single-letter code). N-Boc propargylamine was treated with bromine in the presence of base to afford the 1-bromo derivative **6**, which was subjected to acid deprotection to give **7** and subsequent HATU-mediated coupling with the appropriate amino acid. Because polar amino acids proved difficult to isolate and purify, apolar protecting groups were employed to facilitate isolation and purification for serine (**PLS**) and threonine (**PLT**), as well as for charged amino acids aspartic acid (**PLD**), glutamic acid (**PLE**), lysine (**PLK**), arginine (**PLR**), and histidine (**PLH**). For the other polar amino acids (**PLN**, **PLQ**, **PLA**, **PLG**, **PLKAc**), the products were subjected to multiple chromatographic purifications without aqueous work-up, which provided the best means of isolating the pure products. Overall, the coupled products were isolated in 9–67% yield. The protected derivatives were subjected to TFA-mediated deprotection to afford the final PepLites in 31–100% yield. Basic PepLites were isolated as the TFA salts.

Although the 1-bromoalkyne moiety is relatively inert, its reactivity has been reported. Indeed, during the course of this work, it was shown that such species are susceptible to reaction with activated cysteines under biologically relevant conditions.^20,21^ This gave rise to concerns that the PepLites may be unstable to thiol species and particularly to reaction with dithiothreitol (DTT) in crystallization buffers. Accordingly, we conducted NMR stability studies by incubation of a representative PepLite (PLE) in pH7.4 phosphate buffer in the presence or absence of reducing agents DTT or tris(2-carboxyethyl)phosphine (TCEP) (Figure S1). This revealed that the PepLites are stable in buffer and in the presence of TCEP but, as expected, show appreciable decomposition in the presence of DTT with a half-life of ca. 2 days. Therefore, subsequent crystallization experiments were carried out using TCEP as the reducing agent.

### PepLites Bind to ATAD2 and BRD4

**Figure 8:**
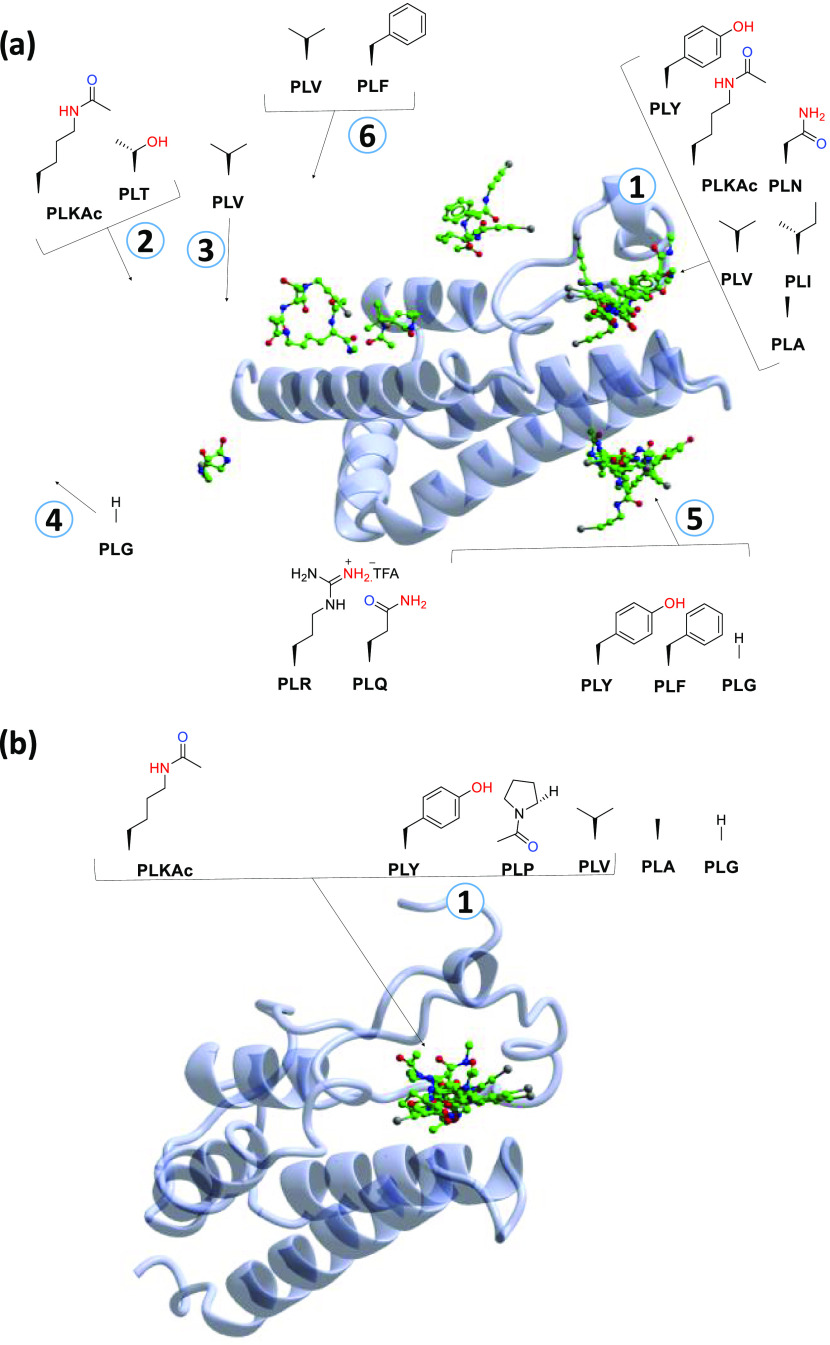
PepLite screening of (a) ATAD2 (PDB: 7R05, 7R0Y, 7Z9H, 7Z9I, 7Z9J, 7Z9N, 7Z9O, 7Z9S, 7Z9U) and (b) BRD4 (PDB: 7ZFU, 7ZFV, 7ZFY, 7ZFZ, 7ZG1, 7ZG2).

To analyze the outcome of the PepLite crystallographic screen, we evaluated both anomalous difference and PanDDA event maps. In most cases, the anomalous signal was not observed; however, the relevant electron density was observed. Of the 22 PepLites screened, 9 showed binding to ATAD2 and 6 to BRD4 (Figure 8). Interestingly, the 6 PepLites bound to BRD4 were all observed to bind in the orthosteric site, whereas ATAD2 showed PepLite binding events distributed between the orthosteric site and allosteric site 6 identified from the FragLite screening.

**Figure 9:**
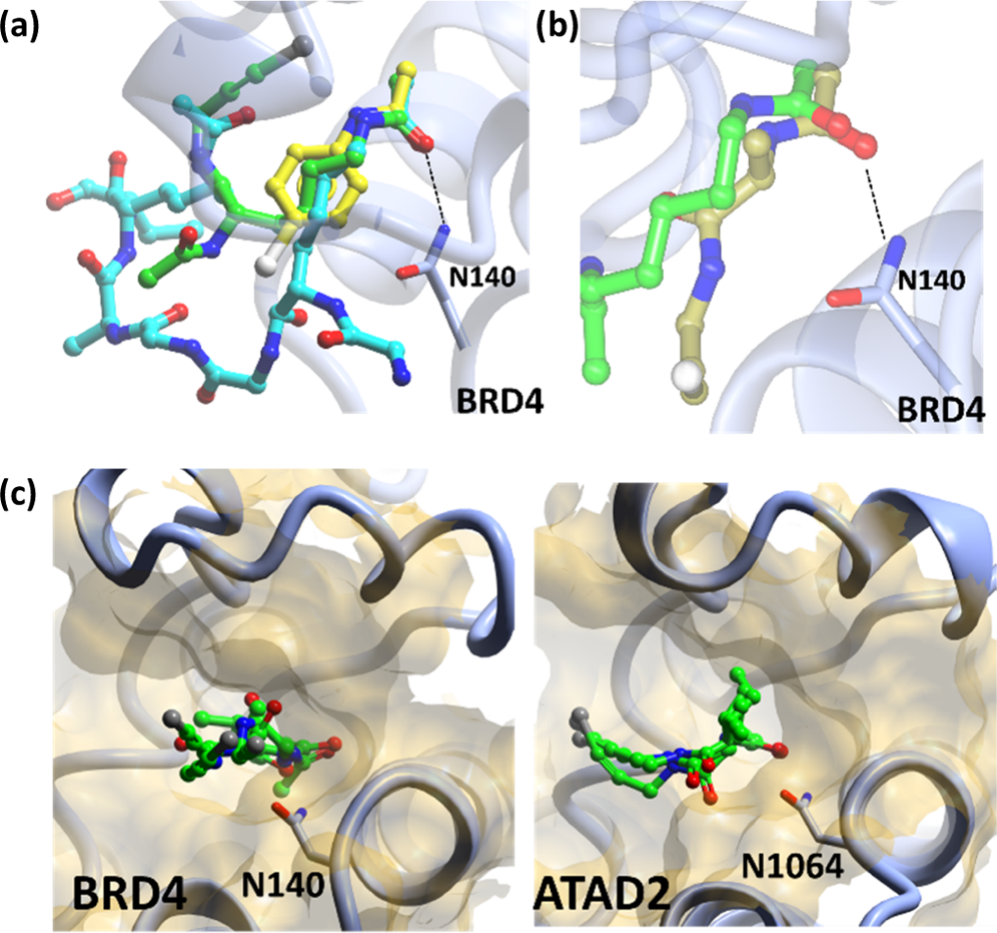
Interactions of the PepLites within the acetyl lysine pocket of ATAD2 and BRD4. (a) **PLKAc** (green, PDB: 7ZG2) overlaid with diacetylated histone peptide (cyan, PDB: 3UVX) and **FL11** (yellow, PDB: 7ZAA) bound to BRD4; (b) **PLA** (gold, PDB: 7ZFV) and **PLKAc** (green, PDB: 7ZG2) bound to BRD4. (c) **PLG**, **PLA**, **PLV** bound to BRD4 (PDB: 7ZFY, 7ZFV, 7ZFZ) and **PLA**, **PLV**, **PLI** bound to ATAD2 (PDB: 7Z9I, 7Z9N, 7R05).

The acetyl group of PepLite **PLKAc** made identical contacts with Asn140 in BRD4 to those of the acetyl groups of an acetyl lysine peptide and **FL11** (Figure 9a), thus identifying the dominant recognition interaction. A similar binding position was observed for the amide nitrogen and its adjacent carbon atom for all three of these ligands, although the location of atoms beyond this point was divergent. With the PepLite scaffold mimicking the canonical acetylated lysine binding interaction with Asn140, the side chain is orientated toward the lid of the binding cleft as observed with **PLA** (Figure 9b). BRD4 has a relatively enclosed and reduced volume acetyl lysine binding cleft in comparison to ATAD2, this observation in part formed the basis of the reduced druggability of ATAD2.^22^ As with BRD4, the acetylated PepLite scaffold formed the same interaction with ATAD2 and conserved Asn1064 as that of acetylated lysine histone. However, in the case of ATAD2, there were an increased number of binding events of lipophilic groups that point toward the lid of the binding cleft, **PLA**, **PLV**, and **PLI** compared to **PLA** for BRD4 (Figure 9c) (Note: **PLG**, **PLV**, **PLP**, and **PLY** do not engage with the Asn140 of BRD4 through the same interaction). This observation is indicative of the larger binding cleft in ATAD2 and the propensity of BRD4 to accommodate multiple chemical scaffolds.

The 1-bromoalkyne moiety of PepLite scaffold was identified as an electrophile targeting cysteines during a crystallographic screen against MPro.^21^ The formation of similar covalent adducts were observed against ATAD2 through a disulfide bridge near the base of the acetyl lysine binding pocket (Cys1057–Cys1079) with PepLites **PLR**, **PLQ**, **PLY**, and **PLG** (Figure 8a) the site was identified as site 6 from the FragLite screen (Figure 3a). This disulfide site has been shown to influence the substrate recognition of ATAD2, and could potentially present as a site for irreversible allosteric inhibition of the bromodomain.^23^

### Lead Identification from FragLites

**Figure 10:**
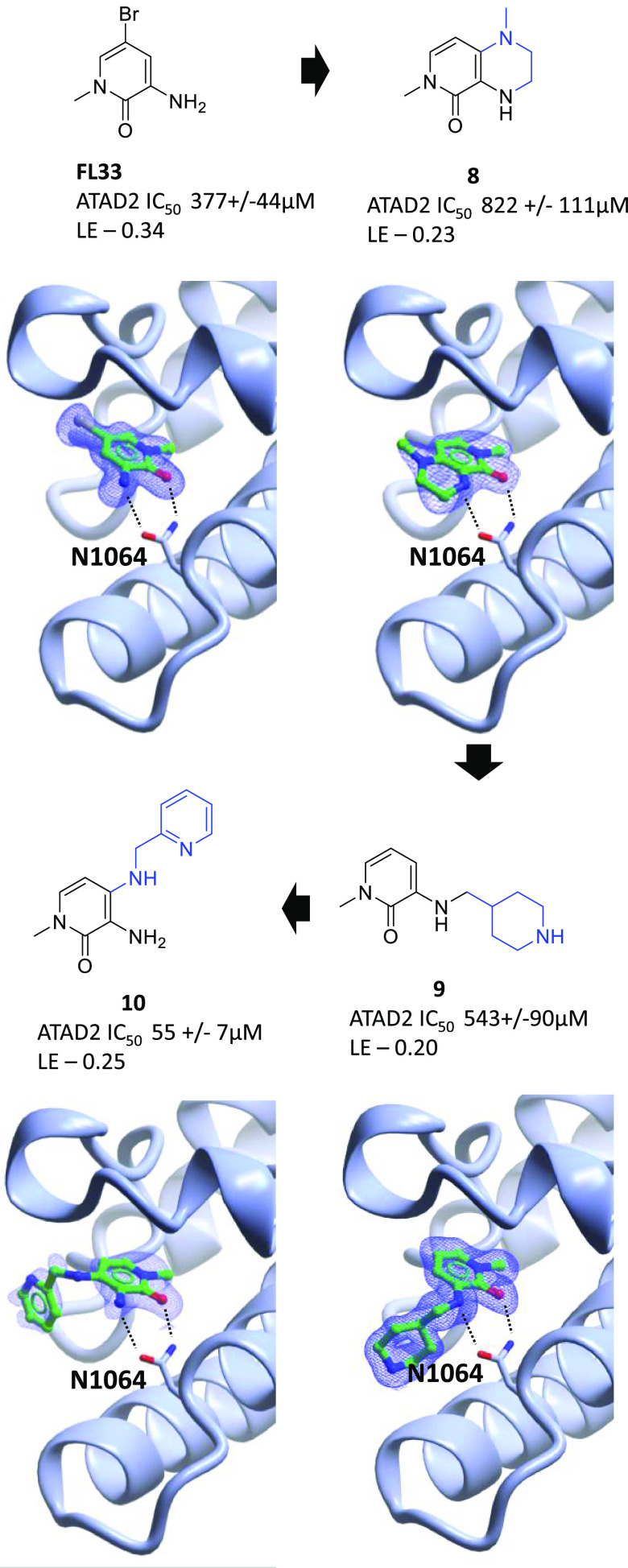
Follow-up of FL33 against ATAD2.

For FragLite hits to play a role as start points for chemical probe and drug discovery, it is important that their chemistry and binding modes should be compatible with elaboration to generate biologically active compounds. Although we have previously demonstrated this to be the case for hits against CDK2, we attempted to demonstrate the generality of the approach here by conducting limited medicinal chemistry around ATAD2 hit **FL33** (Figure 10). Iterative medicinal chemistry was undertaken, informed by the constellation of FragLites that shared binding to the orthosteric site. **FL33** adopts a binding mode that reflects interactions made by the acetyl lysine natural ligand, while other orthosteric site-binding ligands, including the scaffold of the PepLites, demonstrate the availability of space adjacent to the pyridone 4-position of **FL33**. Comparison of the PepLite binding modes implied a suitable region in which substitution would be tolerated. Accordingly, introduction of a fused piperazine at the 3- and 4-positions yielded **8** that retained binding to ATAD2 and offers further vectors for elaboration toward potentially productive interactions. Analysis of the crystal structures suggested that further substitution of the 3- and 4-positions would be tolerated. Accordingly, 3-piperidinylmethylamino derivative **9** retained potency and a ca. 10-fold increase in affinity was demonstrated with the 4-pyridinylmethyl derivative **10** (HTRF peptide-displacement assay). Hence, this shows that the FragLite hits can be developed in short order, using information from the overall FragLite binding map to compounds with leadlike potency.

## Discussion

The high hit rate for FragLite compounds in crystallographic soaking experiments confirms that FragLite mapping can identify binding sites of different character (cofactor binding site of CDK2,^5^ peptide-binding site of the bromodomains, sites remote from the orthosteric site in all cases) on different underlying protein scaffolds.

FragLite mapping of bromodomains from ATAD2 and BRD4 compared two members of a common structural family. FragLites identify the orthosteric site of both family members. The hit rate of FragLites at the orthosteric site of BRD4 is higher than at that of ATAD2. This finding is consistent with computational predictors of the relative druggability of the two sites.^14^ Our findings also correlate with anecdotal perception of the relative tractability of the two domains, i.e., the amount of work needed to achieve high-affinity inhibitors of each protein. It is believed that the increased druggability of BRD4 is due, at least in part, to ligands forming lipophilic interactions with the “WPF shelf”; our results suggest that the KAc binding site in BRD4 is also more amenable to binding small-molecule ligands than that of ATAD2. The similar frequencies of FragLites populating allosteric sites in both proteins suggest that their potential to bind allosteric ligands is similar. While further FragLite campaigns, applied in advance of fuller drug discovery programs, will be needed to turn this subjective impression into a statistically supported view, the findings presented here show that FragLite mapping can provide an initial step in experimentally determining the relative druggability of a particular protein target.

Detection of the FragLites for bromodomains from ATAD2 and BRD4 could be achieved through either inspection of the anomalous difference electron density maps or, with equal effectiveness, through application of the PanDDA algorithm to the diffraction datasets. This differs slightly from the previously reported CDK2 FragLite campaign, where anomalous scattering proved to be more sensitive as an approach for identifying hits. The comparable performance of PanDDA in this case may relate to its superior performance in the analysis of the bromodomain events or to a relatively weak anomalous signal generated in these crystal systems. If the latter, then factors that may be at play include the relative ordering of the bound compounds (a function of the specific ligand recognition events), relatively higher photolysis or damage-induced loss of bromine (a function of the radiative dose absorbed by the ATAD2 and BRD4 crystals), or details of the multiplicity of the datasets collected. Evaluation of many more FragLite campaigns will be needed to understand where the anomalous scattering signal can contribute most effectively, and the implications this may have for crystallization and data collection strategies. The combined application of PanDDA event maps and anomalous difference maps provides the shortest route to building a reliable model for a bound ligand, and there is scope for algorithmic development to automate the process of ligand fitting to exploit both sources of information.

This study significantly increases the count of FragLite binding events observed to date and allows us to begin to statistically evaluate their performance against their design principles. As intended, FragLites demonstrate a mixture of hydrophobic and hydrogen-bonding interactions, with an average of >1 hydrogen-bonding contact per compound. We have also established that their binding is not dominated by crystal contacts, thereby confirming the utility of FragLite mapping as a tool to predict the location of solution-phase binding events, in particular productive hydrogen-bonding interactions.

We have further shown that sites identified in a relatively efficient and inexpensive FragLite campaign recapitulate those identified in a larger screening campaign with a well-formulated and mature fragment library. Of course, the fuller fragment campaign yielded substantially more hits, which is desirable for identifying the optimal start point for a drug or chemical probe discovery program. We suggest that FragLites can therefore play a number of roles in the fragment screening environment. First, they can provide a sensitive and effective pre-screen of ligandability, where 40 or 50 datasets can provide a guide as to the likely utility of a larger and more expensive campaign. Second, in some settings, they may provide sufficient information to commence hit-to-lead chemistry, especially if high-throughput fragment screening is challenging. Finally, they represent highly valuable components of a larger crystallographic screen, and we anticipate that this is where they will fit into most drug discovery programs. To further support the utility of FragLite hits as seeds for such programs, we have again demonstrated that FragLites lend themselves to ready elaboration to generate compounds that are of sufficiently high affinity to show activity in a biological assay, and therefore amenable to further optimization.

PepLites extend the family of FragLites by including small molecules with substantially different chemistry and geometry, which return high hit rates in crystallographic screening. The alkynyl bromide group, introduced to get around stability issues with alkyl halide bonds, appears to be a biocompatible structure that can provide a halogen beacon in anomalous scattering maps. The peak heights for the bromine positions in PepLite binding events were, however, lower on average than those observed for FragLites. This difference may reflect either greater loss of bromine atoms due to photolysis or secondary X-ray damage events, or less well-ordered binding of the extended bromoalkyne moiety.

The clear binding of **PLKAc** demonstrates that an appropriate PepLite can bind in a pose that recapitulates the biologically relevant binding mode. Notably, however, despite marked 3D character and a relatively high rotatable bond count, PepLites are also able to adopt druglike binding modes even when they don’t recapitulate interactions of authentic protein partners.

PepLites showed a higher hit rate than FragLites in the orthosteric site of ATAD2. We hypothesize that this is due to their size and 3D character, which may be more compatible with the more “open” character of the ATAD2 peptide-binding groove. Thus, PepLites may be preferred scaffolds in certain active sites, where FragLites and related compounds are more compatible with others. Therefore, the incorporation of the PepLite amino acid scaffold into the FragLite screening library has improved the capacity of the combined library to find productive binding modes against a broader range of targets.

## Conclusions

This work demonstrates the use of FragLites and PepLites in establishing druggability, illustrated by two bromodomain proteins. FragLite mapping is comparable to a full fragment screen in identifying ligand binding sites on proteins. Analysis of FragLite binding identifies critical hydrogen bonding interactions that can be exploited to generate compounds of leadlike potency and could guide further hit generation activities. Hence, this shows that FragLites and PepLites could be used in isolation or in conjunction with other hit-finding activities to develop drug candidates or chemical probes for novel targets.

## Experimental Section

### General Information

Chemicals were purchased from commercial suppliers and used without further purification. Thin-layer chromatography (TLC) was performed on aluminum plates coated with 60 F_254_ silica from Merck. Flash chromatography was carried out using a Biotage SP4, Biotage Isolera, or Varian automated flash system with Silicycle or GraceResolve normal-phase silica gel pre-packed columns. Fractions were collected at 254 nm or, if necessary, on all wavelengths between 200 and 400 nm. Microwave irradiation was performed in a Biotage Initiator Sixty in sealed vials. Reactions were irradiated at 2.45 GHz and were able to reach temperatures between 60 and 250 °C. Heating was at a rate of 2–5 °C/s, and the pressure was able to reach 20 bar. Final compound purity is >95% determined by HPLC or NMR.

### Analytical Equipment

Melting points were measured using a Stuart automatic melting point SMP40 apparatus. Fourier transform infrared (FTIR) spectra were measured using an Agilent Cary 630 FTIR. The abbreviations for peak description are as follows: b = broad; w = weak; m = medium and s = strong. Ultraviolet (UV) spectra were recorded on a Hitachi U-2900 spectrophotometer in ethanol. High-resolution mass spectrometry (HRMS) was provided by the ESPRC National Mass Spectrometry Service, University of Wales, Swansea, or conducted using an Agilent 6550 iFunnel QTOF LCMS with an Agilent 1260 Infinity UPLC system. The sample was eluted on Acquity UPLC BEH C18 (1.7 μm, 2.1 × 50 mm^2^) with a flow rate of 0.7 mL/min and run at a gradient of 1.2 min 5–95% 0.1% aq. HCOOH in MeCN.

LCMS analyses were conducted using a Waters Acquity UPLC system with photodiode array (PDA) and evaporating light scattering detector (ELSD). When a 2 min gradient was used, the sample was eluted on an Acquity UPLC BEH C18, 1.7 μm, 2.1 × 50 mm^2^, with a flow rate of 0.6 mL/min using 5–95% 0.1% HCOOH in MeCN. Analytical purity of compounds was determined using Waters XTerra RP18, 5 μm (4.6 × 150 mm^2^) column at 1 mL/min using either 0.1% aq. ammonia and MeCN or 0.1% aq. HCOOH and MeCN with a gradient of 5–100% over 15 min.

^1^H NMR spectra were obtained using a Bruker Avance III 500 spectrometer using a frequency of 500 MHz. ^13^C spectra were acquired using the Bruker Avance III 500 spectrometer operating at a frequency of 126 MHz. The abbreviations for spin multiplicity are as follows: s = singlet; d = doublet; t = triplet; q = quartet, p = quintuplet, h = sextuplets, and m = multiplet. Combinations of these abbreviations are employed to describe more complex splitting patterns (e.g., dd = doublet of doublets).

### General Procedure: PepLite Synthesis

HATU (1.5 equiv), DIPEA (3.0 equiv), and the acid starting material (1.5 equiv) were dissolved in DMF (3–6 mL) and stirred together at rt for 10 min. 3-Bromoprop-2-yn-1-amine hydrochloride was added, and the reaction mixture was stirred at 40 °C overnight. The reaction mixture was allowed to cool to rt, diluted with EtOAc or DCM, and washed with saturated aqueous sodium bicarbonate solution, brine, and water. The organic layer was dried over MgSO_4_, filtered, and evaporated. The crude product was then purified by either normal- or reversed-phase chromatography.

#### tert-Butyl(3-bromoprop-2-yn-1-yl)carbamate

A solution of KOH (2.7 g, 48 mmol) in water (15 mL) was added dropwise to a solution of N-boc propargylamine (3 g, 19 mmol) in MeOH (45 mL) at 0 °C under nitrogen. The resulting solution was stirred at 0 °C for 10 min, then bromine (1.1 mL, 21 mmol) was added dropwise. The reaction mixture was allowed to warm to rt, stirred for 24 h, diluted with water, and extracted with diethyl ether. The organic extracts were combined, dried over MgSO_4_, and evaporated. The crude product was purified by flash silica chromatography, elution gradient 0–10% EtOAc in petrol, to afford tert-butyl(3-bromoprop-2-yn-1-yl)carbamate (3.5 g, 79%) as a white solid.

*R*_f_ = 0.34 (10% EtOAc in petrol); m.p. 108–110 °C; IR ν_max_ (cm^–1^) 3345, 2982, 2219, 2121, 2082; ^1^H NMR (500 MHz, DMSO-*d*_6_) δ 1.39 (s, 9H), 3.76 (d, *J* = 5.9 Hz, 2H), 7.30 (d, *J* = 6.1 Hz, 1H); ^13^C NMR (126 MHz, DMSO-*d*_6_) δ 28.6, 30.9, 43.4, 78.5, 78.8, 155.7; LCMS (ESI^+^) *m*/*z* = 133.9 [M-Boc + H]^+^; HRMS calc’d for C_8_H_12_^79^BrNO_2_ [M + Na]^+^ 255.9949 found 256.0209.

#### 3-Bromoprop-2-yn-1-amine hydrochloride

tert-Butyl(3-bromoprop-2-yn-1-yl)carbamate (1.1 g, 4.7 mmol) was dissolved in 4 M HCl in dioxane (30 mL). The reaction mixture was stirred at rt for 2 h and then evaporated to dryness to afford 3-bromoprop-2-yn-1-amine hydrochloride (0.79 g, 99%) as a yellow solid.

m.p. 169–172 °C; IR ν_max_ (cm^–1^) 2856, 2629, 2226, 2121, 2074; ^1^H NMR (500 MHz, DMSO-*d*_6_) δ 3.78 (s, 2H), 8.48 (s, 3H); ^13^C NMR (126 MHz, DMSO-*d*_6_) δ 29.7, 49.4, 73.9; LCMS (ESI^+^) *m*/*z* = 134.0 [M + H]^+^; HRMS calcd for C_3_H_5_^79^BrN 133.9605 [M + H]^+^ found 133.9598.

#### (S)-2-Acetamido-N-(3-bromoprop-2-yn-1-yl)-3-phenylpropanamide (PLF)

Synthesized according to the general procedure using N-acetyl-l-phenylalanine (184 mg, 0.89 mmol). The crude product was purified by flash silica chromatography, elution gradient 0–100% EtOAc in petrol, and SCX chromatography, using eluents 10% MeOH/DCM and 10% 6 M NH_3_/MeOH, to afford (S)-2-acetamido-N-(3-bromoprop-2-yn-1-yl)-3-phenylpropanamide (30 mg, 16%) as an off-white solid.

*R*_f_ = 0.35 (70% EtOAc in petrol); mp: 158–164 °C; IR ν_max_ (cm^–1^) 3261, 3081, 2928, 2855, 2219, 2114; ^1^H NMR (500 MHz, Methanol-*d*_4_) δ 1.81 (s, 3H), 2.72–3.03 (m, 2H), 3.76–3.88 (m, 2H), 4.45 (dd, *J* = 8.5, 6.5 Hz, 1H), 7.07–7.15 (m, 3H), 7.13–7.21 (m, 2H); ^13^C NMR (126 MHz, Methanol-*d*_4_) δ 21.0, 29.0, 37.6, 41.8, 54.7, 75.3, 126.4, 128.0, 128.9, 136.8, 171.7, 171.9; LCMS (ESI^+^) *m*/*z* = 325.2 [M + H]^+^; calcd for C_14_H_15_^79^BrN_2_O_2_ 345.0215 [M + Na]^+^ found 345.0304.

#### (S)-2-Acetamido-N-(3-bromoprop-2-yn-1-yl)-3-(1H-indol-3-yl)propanamide (PLW)

Synthesized according to the general procedure using N-acetyl-l-tryptophan (433 mg, 1.8 mmol). The crude product was purified by flash silica chromatography, elution gradient 0–10% MeOH in DCM, to afford (S)-2-acetamido-N-(3-bromoprop-2-yn-1-yl)-3-(1H-indol-3-yl)propanamide (170 mg, 40%) as an off-white solid.

*R*_f_ = 0.45 (10% MeOH in DCM); m.p. 160–164 °C; UV λ_max_ (EtOH/nm) 282.6, 222.2; IR ν_max_ (cm^–1^) 3247, 3074, 2914, 2223, 2113; ^1^H NMR (500 MHz, Methanol-*d*_4_) δ 1.92 (s, 3H), 3.06–3.26 (m, 2H), 3.89 (d, *J* = 4.0 Hz, 2H), 4.61 (t, *J* = 7.0 Hz, 1H), 7.00–7.04 (m, 1H), 7.07–7.10 (m, 2H), 7.31–7.34 (m, 1H), 7.57–7.59 (m, 1H); ^13^C NMR (126 MHz, Methanol-*d*_4_) δ 21.1, 27.6, 29.0, 54.3, 109.4, 110.9, 117.9, 118.4, 121.0, 123.1, 127.4, 171.7, 172.5, C_q_ absent; LCMS (ESI+) *m*/*z* = 364.2 [M + H]^+^; HRMS calcd for C_16_H_17_^79^BrN_3_O_2_ 362.0504 [M + H]^+^ found 362.0589.

#### (S)-2-Acetamido-N-(3-bromoprop-2-yn-1-yl)-4-methylpentanamide (PLL)

Synthesized according to the general procedure using N-acetyl-l-leucine (68 mg, 0.39 mmol). The crude product was purified by flash silica chromatography, elution gradient 0–70% EtOAc in petrol, to afford (S)-2-acetamido-N-(3-bromoprop-2-yn-1-yl)-4-methylpentanamide (18 mg, 11%) as a crystalline white solid.

*R*_f_ = 0.38 (70% EtOAc in petrol); m.p. 149–153 °C; IR ν_max_ (cm^–1^) 3258, 3067, 2923, 2868, 2223, 2112; ^1^H NMR (500 MHz, Methanol-*d*_4_) δ 0.97 (dd, *J* = 18.3, 6.6 Hz, 6H), 1.56–1.66 (m, 2H), 1.71 (dh, *J* = 7.9, 6.6 Hz, 1H), 2.00 (s, 3H), 4.20–4.36 (m, 2H), 4.43 (dd, *J* = 9.3, 5.9 Hz, 1H); ^13^C NMR (126 MHz, Methanol-*d*_4_) δ 20.6, 21.0, 22.0, 24.6, 40.7, 42.8, 51.8, 104.5, 122.4, 171.9, 173.7; LCMS (ESI+) *m*/*z* = 289.1 [M + H]^+^; HRMS calcd for C_11_H_18_^79^BrN_2_O_2_ 289.0551 [M + H]^+^ found 289.0708.

#### (2S,3R)-2-Acetamido-N-(3-bromoprop-2-yn-1-yl)-3-methylpentanamide (PLI)

Synthesized according to the general procedure using N-acetyl-l-isoleucine (154 mg, 0.89 mmol). The crude product was purified by flash silica chromatography, elution gradient 0–100% EtOAc in petrol, to afford (2S,3R)-2-acetamido-N-(3-bromoprop-2-yn-1-yl)-3-methylpentanamide (133 mg, 74%) as a white solid.

*R*_f_ = 0.35 (70% EtOAc in petrol); mp: 158–163 °C; IR ν_max_ (cm^–1^) 3274, 3071, 2963, 2930, 2224, 2118; 1H NMR (500 MHz, DMSO-*d*_6_) δ 0.78–0.85 (m, 6H), 1.03–1.14 (m, 1H), 1.23–1.45 (m, 1H), 1.65–1.80 (m, 1H), 1.86 (d, *J* = 12.3 Hz, 3H), 3.83–3.95 (m, 2H), 4.10–4.29 (m, 1H), 7.82 (dd, *J* = 46.8, 9.0 Hz, 1H), 8.38 (dt, *J* = 28.4, 5.6 Hz, 1H); ^13^C NMR (126 MHz, DMSO-*d*_6_) δ 11.2, 14.9, 22.5, 25.0, 28.8, 36.6, 43.0, 56.0, 77.3, 169.2, 171.2; LCMS (ESI+) *m*/*z* = 289.1 [M + H]^+^; HRMS calcd for C_11_H_18_^79^BrN_2_O_2_ 289.0551 [M + H]^+^ found 289.0546. NMR analysis indicated a 1:1 mixture of diastereoisomers.

#### (S)-2-Acetamido-N-(3-bromoprop-2-yn-1-yl)-4-(methylthio)butanamide (PLM)

Synthesized according to the general procedure using N-acetyl-l-methionine (253 mg, 1.3 mmol). The crude product was purified by reversed-phase flash chromatography, elution gradient 10–40% acetonitrile (0.1% NH3) in water, to afford (S)-2-acetamido-N-(3-bromoprop-2-yn-1-yl)-4-(methylthio)butanamide (50 mg, 17%) as a white solid.

*R*_f_ = 0.60 (40% ACN (0.1% NH_3_) in water); mp: 128–134 °C; IR ν_max_ (cm^–1^) 3272, 3073, 2914, 2224, 2119; ^1^H NMR (500 MHz, Methanol-*d*_4_) δ 1.87–1.96 (m, 1H), 2.01 (s, 3H), 2.02–2.10 (m, 1H), 2.11 (s, 3H), 2.46–2.57 (m, 2H), 3.95–4.05 (m, 2H), 4.45 (dd, *J* = 8.8, 5.3 Hz, 1H); ^13^C NMR (126 MHz, Methanol-*d*_4_) δ 13.9, 21.1, 29.1, 29.7, 31.3, 41.7, 52.5, 75.5, 172.1, 172.3; LCMS (ESI+) *m*/*z* = 307.1 [M + H]^+^; HRMS calcd for C_10_H_16_^79^BrN_2_O_2_S 307.0115 [M + H]^+^ found 307.0108.

#### (S)-1-Acetyl-N-(3-bromoprop-2-yn-1-yl)pyrrolidine-2-carboxamide (PLP)

Synthesized according to the general procedure using (S)-1-acetyl-pyrrolidine-2-carboxylic acid (277 mg, 1.8 mmol). The crude product was purified twice by flash silica chromatography using elution gradients 0–10% MeOH in EtOAc and then 0–10% MeOH in DCM. The semipurified crude product was then dissolved in DCM and washed with water. The organic layer was dried over MgSO_4_, filtered, and evaporated to afford (S)-1-acetyl-N-(3-bromoprop-2-yn-1-yl)pyrrolidine-2-carboxamide (52 mg, 17%) as a light brown gum.

*R*_f_ = 0.56 (10% MeOH in DCM); IR ν_max_ (cm^–1^) 3336, 2976, 2928, 2876, 2220, 2116; ^1^H NMR (500 MHz, DMSO-*d*_6_) δ 1.72–1.91 (m, 4H), 1.95–2.23 (m, 3H), 3.33–3.57 (m, 2H), 3.84–3.98 (m, 2H), 4.26 (m, 1H), 8.08–8.56 (m, 1H); ^13^C NMR (126 MHz, DMSO-*d*_6_) δ 22.8, 24.7, 29.4, 30.1, 42.3, 48.0, 60.3, 77.8, 169.0, 172.2; LCMS (ESI+) *m*/*z* = 273.1 [M + H]^+^; HRMS calcd for C_10_H_14_^79^BrN_2_O_2_ 273.0238 [M + H]^+^ found 273.0246.

#### (S)-2-Acetamido-N1-(3-bromoprop-2-yn-1-yl)pentanediamide (PLQ)

Synthesized according to the general procedure using (s)-2-acetamido-5-amino-5-oxopentanoic acid (248 mg, 1.3 mmol) and evaporating the reaction mixture to afford the crude product without aqueous work-up. The crude product was purified three times by flash silica chromatography, elution gradients 0–15% MeOH in DCM, to afford (S)-2-acetamido-N1-(3-bromoprop-2-yn-1-yl)pentanediamide (22 mg, 8%) as a white solid.

*R*_f_ = 0.30 (15% MeOH in EtOAc); IR ν_max_ (cm^–1^) 3280, 3212, 3070, 2922, 2356, 2117, 1993; ^1^H NMR (500 MHz, Methanol-*d*_4_) δ 1.84–1.93 (m, 1H), 2.00 (s, 3H), 2.08 (m, 1H), 2.29 (t, *J* = 7.7 Hz, 2H), 3.99 (s, 2H), 4.32 (dd, *J* = 9.1, 5.1 Hz, 1H); ^13^C NMR (126 MHz, Methanol-*d*_4_) δ 22.5, 28.9, 30.5, 32.5, 43.3, 54.2, 76.8, 173.5, 173.6, 177.7; LCMS (ESI+) *m*/*z* = 326.1 [M + Na]^+^; HRMS calcd for C_10_H_15_^79^BrN_3_O_3_ 304.0296 [M + H]^+^ found 304.0294.

#### (S)-2-Acetamido-N-(3-bromoprop-2-yn-1-yl)-3-(tert-butoxy)propanamide

Synthesized according to the general procedure using N-acetyl-O-tert-butyl-l-serine (350 mg, 2.1 mmol). The crude product was purified by flash silica chromatography, elution gradient 0–10% MeOH in DCM, to afford (S)-2-acetamido-N-(3-bromoprop-2-yn-1-yl)-3-(tert-butoxy)propanamide (277 mg, 45%) as a colorless gum, which crystallized on standing.

*R*_f_ = 0.56 (10% MeOH in DCM); mp: 86–89 °C; IR ν_max_ (cm^–1^) 3266, 2975, 2927, 2878, 2220; ^1^H NMR (500 MHz, Methanol-*d*_4_) δ 1.21 (s, 9H), 2.03 (s, 3H), 3.56–3.70 (m, 2H), 4.01 (d, *J* = 1.1 Hz, 2H), 4.42 (t, *J* = 5.1 Hz, 1H); ^13^C NMR (126 MHz, Methanol-*d*_4_) δ 21.1, 26.2, 29.0, 54.0, 61.4, C_q_ absent; LCMS (ESI+) *m*/*z* = 263.1 [M-tBu + H]^+^; HRMS calcd for C_12_H_20_^79^BrN_2_O_3_ 319.0657 [M + H]^+^ found 319.1485.

#### (S)-2-Acetamido-N-(3-bromoprop-2-yn-1-yl)-3-hydroxypropanamide (PLS)

(S)-2-Acetamido-N-(3-bromoprop-2-yn-1-yl)-3-(tert-butoxy)propanamide (42 mg, 0.13 mmol) was dissolved in anhydrous DCM (10 mL) and TFA (5 mL) and 0 °C under nitrogen. The reaction mixture was allowed to warm to rt, stirred for 3 h, and then evaporated to dryness. The crude product was purified by flash silica chromatography, elution gradient 0–10% MeOH in DCM, to afford (S)-2-acetamido-N-(3-bromoprop-2-yn-1-yl)-3-hydroxypropanamide in quantitative yield as a white solid.

*R*_f_ = 0.33 (10% MeOH in DCM); mp: 157–158 °C; IR ν_max_ (cm^–1^) 3320, 2937, 2223, 2119; ^1^H NMR (500 MHz, Methanol-*d*_4_) δ 2.04 (s, 3H), 3.74–3.82 (m, 2H), 3.97–4.07 (m, 2H), 4.42 (t, *J* = 5.4 Hz, 1H); ^13^C NMR (126 MHz, Methanol-*d*_4_) δ 21.2, 29.1, 41.8, 55.4, 61.6, 75.4, 170.9, 172.1; LCMS (ESI+) *m*/*z* = 263.1 [M + H]^+^; HRMS calcd for C_8_H_12_^79^BrN_2_O_3_ 263.0031 [M + H]^+^ found 263.0026.

#### (2S,3S)-2-Acetamido-N-(3-bromoprop-2-yn-1-yl)-3-(tert-butoxy)butanamide

Synthesized according to the general procedure using (2S,3R)-2-acetamido-3-(tert-butoxy)butanoic acid (406 mg, 1.9 mmol). The crude product was purified by flash silica chromatography, elution gradient 0–10% MeOH in DCM, to afford (2S,3S)-2-acetamido-N-(3-bromoprop-2-yn-1-yl)-3-(tert-butoxy)butanamide (198 mg, 42%) as a white solid.

*R*_f_ = 0.46 (10% MeOH in DCM); mp: 180–183 °C; IR ν_max_ (cm^–1^) 3271, 3078, 2969, 2935, 2222, 2113; ^1^H NMR (500 MHz, Methanol-*d*_4_) δ 1.16 (d, *J* = 6.2, Hz, 3H), 1.21 (s 9H), 2.01 (s, 3H), 3.91–4.09 (m, 3H, H7), 4.32 (d, *J* = 7.5 Hz, 1H); ^13^C NMR (126 MHz, Methanol-*d*_4_) δ 18.6, 21.2, 27.3, 28.9, 41.9, 58.8, 67.2, 74.2, 75.6, 171.2, 171.9; LCMS (ESI+) *m*/*z* = 333.2 [M + H]^+^; HRMS calcd for C_13_H_22_^79^BrN_2_O_3_ 333.0813 [M + H]^+^ found 333.0808.

#### (2S,3S)-2-Acetamido-N-(3-bromoprop-2-yn-1-yl)-3-hydroxybutanamide (PLT)

(2S,3S)-2-Acetamido-N-(3-bromoprop-2-yn-1-yl)-3-(tert-butoxy)butanamide (80 mg, 0.24 mmol) was dissolved in anhydrous DCM (20 mL) and TFA (10 mL) at 0 °C under nitrogen. The reaction mixture was allowed to warm to rt, stirred for 3 h, and then evaporated to dryness. The crude product was purified by flash silica chromatography, elution gradient 0–15% MeOH in DCM, to afford (2S,3S)-2-acetamido-N-(3-bromoprop-2-yn-1-yl)-3-hydroxybutanamide (38 mg, 57%) as a white solid.

*R*_f_ = 0.34 (10% MeOH in DCM); mp: 189–192 °C; IR ν_max_ (cm^–1^) 3280, 3085, 2973, 2924, 2225, 2115; ^1^H NMR (500 MHz, Methanol-*d*_4_) δ 1.21 (d, *J* = 6.5 Hz, 3H), 2.03 (s, 3H), 3.97–4.06 (m, 3H), 4.33 (d, *J* = 6.5 Hz, 1H); ^13^C NMR (126 MHz, Methanol-*d*_4_) δ 18.2, 21.1, 29.0, 41.8, 58.7, 67.1, 75.4, 170.9, 172.0; LCMS (ESI+) *m*/*z* = 277.1 [M + H]^+^; HRMS calcd for C_9_H_14_^79^BrN_2_O_3_ 277.0187 [M + H]^+^ found 277.0182.

#### (S)-2-Acetamido-N1-(3-bromoprop-2-yn-1-yl)succinamide (PLN)

Synthesized according to the general procedure using (S)-2-acetamido-5-amino-5-oxobutanoic acid (155 mg, 0.89 mmol) and pyridine instead of DIPEA. The reaction mixture was evaporated to afford the crude product without aqueous work-up. The crude product was purified by flash silica chromatography, elution gradients 0–10% MeOH in DCM, to afford (S)-2-acetamido-N1-(3-bromoprop-2-yn-1-yl)succinamide (50 mg, 30%) as a white solid.

*R*_f_ = 0.18 (10% MeOH in DCM); mp: n/a, compound decomposed at 173 °C; IR ν_max_ (cm^–1^) 3421, 3277, 3208, 3072, 2922, 2226, 2116; ^1^H NMR (500 MHz, Methanol-*d*_4_) δ 1.99 (s, 3H), 2.58–2.75 (m, 2H), 3.98 (d, *J* = 1.4 Hz, 2H), 4.71 (dd, *J* = 7.6, 5.7 Hz, 1H); ^13^C NMR (126 MHz, Methanol-*d*_4_) δ 22.6, 30.6, 37.8, 43.1, 51.5, 76.8, 173.0, 173.3, 174.8; LCMS (ESI+) *m*/*z* = 290.2 [M + H]^+^; HRMS calcd for C_9_H_13_^79^BrN_3_O_3_ 290.0140 [M + H]^+^ found 290.2265.

#### (S)-2-Acetamido-N-(3-bromoprop-2-yn-1-yl)propanamide (PLA)

Synthesized according to the general procedure using N-acetyl-l-alanine (173 mg, 1.3 mmol) and evaporating the reaction mixture to afford the crude product without aqueous work-up. The crude product was purified three times by flash silica chromatography, elution gradients 0–15% MeOH in DCM and 0–5% MeOH in EtOAc, to afford (S)-2-acetamido-N-(3-bromoprop-2-yn-1-yl)propanamide (28 mg, 13%) as a white solid.

*R*_f_ = 0.33 (5% MeOH in EtOAc); mp: 152–155 °C; IR ν_max_ (cm^–1^) 3269, 3091, 2919, 2413, 2216, 2111; ^1^H NMR (500 MHz, Methanol-*d*_4_) δ 1.32 (d, *J* = 7.2 Hz, 3H), 1.98 (s, 3H), 3.98 (s, 2H), 4.30 (q, *J* = 7.2 Hz, 1H); ^13^C NMR (126 MHz, Methanol-*d*_4_) δ 18.0, 22.4, 30.5, 43.1, 50.3, 76.9, 173.2, 174.9; LCMS (ESI+) *m*/*z* = 249.1 [M + H]^+^; HRMS calcd for C_8_H_12_^79^BrN_2_O_2_ 247.0082 [M + H]^+^ found 247.0082.

#### 2-Acetamido-N-(3-bromoprop-2-yn-1-yl)acetamide (PLG)

Synthesized according to the general procedure using N-acetylglycine (77 mg, 0.66 mmol) and pyridine instead of DIPEA. The reaction mixture was evaporated to afford the crude product without aqueous work-up. The crude product was purified twice by flash silica chromatography, elution gradients 0–10% MeOH in EtOAc, to afford 2-acetamido-N-(3-bromoprop-2-yn-1-yl)acetamide (55 mg, 53%) as a white solid.

*R*_f_ = 0.40 (10% MeOH in DCM); mp: 147–149 °C; IR ν_max_ (cm^–1^) 3298, 3206, 3058, 2930, 2863, 2225, 2111; ^1^H NMR (500 MHz, Methanol-*d*_4_) δ 2.01 (s, 3H), 3.83 (s, 2H), 4.00 (s, 2H); ^13^C NMR (126 MHz, Methanol-*d*_4_) δ 22.5, 30.4, 43.2, 43.4, 76.9, 171.5, 173.9; LCMS (ESI+) *m*/*z* = 233.1 [M + H]^+^. HRMS calcd for C_7_H_10_^79^BrN_2_O_2_ 232.9925 [M + H]^+^ found 232.992.

#### tert-Butyl (S)-3-acetamido-4-((3-bromoprop-2-yn-1-yl)amino)-4-oxobutanoate

Synthesized according to the general procedure using acetyl-l-aspartic acid β-tert-butyl ester (611 mg, 2.6 mmol). The crude product was purified by flash silica chromatography, elution gradient 0–15% MeOH in DCM, to afford tert-butyl (S)-3-acetamido-4-((3-bromoprop-2-yn-1-yl)amino)-4-oxobutanoate (277 mg, 45%) as a colorless gum, which crystallized on standing.

*R*_f_ = 0.45 (10% MeOH in DCM); mp: 127–134 °C; IR ν_max_ (cm^–1^) 3297, 3063, 2981, 2930, 2223; ^1^H NMR (500 MHz, Methanol-*d*_4_) δ 1.45 (s, 9H), 1.99 (s, 3H), 2.57 (dd, *J* = 16.1, 7.7 Hz, 1H), 2.76 (dd, *J* = 16.1, 6.1 Hz, 1H), 3.98 (s, 2H), 4.71 (dd, *J* = 7.7, 6.1 Hz, 1H); ^13^C NMR (126 MHz, Methanol-*d*_4_) δ 22.6, 28.3, 30.6, 38.5, 43.2, 51.3, 76.9, 82.5, 171.2, 172.7, 173.2; LCMS (ESI+) *m*/*z* = 291.2 [M-tBu + H]^+^; HRMS calcd for C_13_H_19_^81^BrN_2_O_4_ 371.0406 [M + Na]^+^ found 371.0460

#### (S)-3-Acetamido-4-((3-bromoprop-2-yn-1-yl)amino)-4-oxobutanoic acid (PLD)

tert-Butyl (S)-3-acetamido-4-((3-bromoprop-2-yn-1-yl)amino)-4-oxobutanoate (180 mg, 0.52 mmol) was dissolved in anhydrous DCM (20 mL) and TFA (10 mL) and 0 °C under nitrogen. The reaction mixture was allowed to warm to rt, stirred for 5 h, and then evaporated to dryness. The crude product was purified by flash silica chromatography, elution gradient 0–10% MeOH in DCM, to afford (S)-3-Acetamido-4-((3-bromoprop-2-yn-1-yl)amino)-4-oxobutanoic acid (97 mg, 67%) as a white solid.

*R*_f_ = 0.11 (10% MeOH in DCM); mp: 138 °C (decomp.); IR ν_max_ (cm^–1^) 3275, 3070, 2919, 2224, 2114, 2081, 1994, 1911; ^1^H NMR (500 MHz, Methanol-*d*_4_) δ 1.99 (s, 3H), 2.65–2.87 (m, 2H), 3.98 (d, *J* = 3.4 Hz, 2H), 4.72 (dd, *J* = 7.5, 5.8 Hz, 1H); ^13^C NMR (126 MHz, Methanol-*d*_4_) δ 22.5, 30.6, 36.8, 43.1, 51.2, 76.8, 172.9, 173.4, 173.8; LCMS (ESI+) *m*/*z* = 291.2 [M + H]^+^; HRMS calcd for C_9_H_12_^79^BrN_2_O_4_ 312.9800 [M + Na]^+^ found 312.9922.

#### tert-Butyl (S)-4-acetamido-5-((3-bromoprop-2-yn-1-yl)amino)-5-oxopentanoate

Synthesized according to the general procedure using acetyl-l-glutamic acid γ-tert-butyl ester (648 mg, 2.6 mmol). The crude product was purified by flash silica chromatography, elution gradient 0–15% MeOH in DCM, to afford tert-butyl (S)-4-acetamido-5-((3-bromoprop-2-yn-1-yl)amino)-5-oxopentanoate (426 mg, 67%) as a colorless gum.

*R*_f_ = 0.61 (10% MeOH in DCM); IR ν_max_ (cm^–1^) 3285, 3065, 2978, 2934, 2224, 2120; ^1^H NMR (500 MHz, Methanol-*d*_4_) δ 1.45 (s, 9H), 1.80–1.90 (m, 1H), 1.99 (s, 3H), 2.00–2.12 (m, 1H), 2.28–2.33 (m, 2H), 3.94–4.02 (m, 2H), 4.32 (dd, *J* = 8.9, 5.4 Hz, 1H);^13^C NMR (126 MHz, Methanol-*d*_4_) δ 21.0, 26.9, 29.1, 41.8, 52.5, 75.4, 80.4, 172.0, 172.2, 172.4; LCMS (ESI+) *m*/*z* = 305.1 [M-tBu + H]^+^; HRMS calcd for C_14_H_22_^79^BrN_2_O_4_ 361.0762 [M + H]^+^ found 361.0763.

#### (S)-4-Acetamido-5-((3-bromoprop-2-yn-1-yl)amino)-5-oxopentanoic acid (PLE)

tert-Butyl (S)-4-acetamido-5-((3-bromoprop-2-yn-1-yl)amino)-5-oxopentanoate (351 mg, 0.97 mmol) was dissolved in anhydrous DCM (40 mL) and TFA (20 mL) and 0 °C under nitrogen. The reaction mixture was allowed to warm to rt, stirred for 3 h, and then evaporated to dryness. The crude product was purified by flash silica chromatography, elution gradient 0–10% MeOH in DCM, to afford (S)-4-acetamido-5-((3-bromoprop-2-yn-1-yl)amino)-5-oxopentanoic acid (91 mg, 31%) as a white solid.

*R*_f_ = 0.02 (20% MeOH in DCM); mp: 169–173 °C; IR ν_max_ (cm^–1^) 3273, 3088, 2936, 2417, 2223; ^1^H NMR (500 MHz, Methanol-*d*_4_) δ 1.86–1.95 (m, 1H), 2.01 (s, 3H), 2.06–2.14 (m, 1H), 2.36–2.42 (m, 2H), 3.99–4.02 (m, 2H), 4.36 (dd, *J* = 9.0, 5.3 Hz, 1H); ^13^C NMR (126 MHz, Methanol-*d*_4_) δ 21.0, 26.9, 29.0, 29.7, 41.8, 52.6, 75.4, 172.1, 172.2, 174.9; LCMS (ESI+) *m*/*z* = 305.1 [M + H]^+^; HRMS calcd for C_10_H_14_^79^BrN_2_O_4_ 305.0136 [M + H]^+^ found 305.0137.

#### O-tert-Butyl-(S)-(5-acetamido-6-((3-bromoprop-2-yn-1-yl)amino)-6-oxohexyl)carbamate

Synthesized according to the general procedure using (S)-2-acetamido-6-((tert-butoxycarbonyl)amino)hexanoic acid (507 mg, 1.8 mmol). The crude product was purified by reversed-phase chromatography, elution gradient 10–55% ACN (0.1% NH3) in water, to afford tert-butyl (S)-(5-acetamido-6-((3-bromoprop-2-yn-1-yl)amino)-6-oxohexyl)carbamate (238 mg, 50%) as a white solid.

*R*_f_ = 0.45 (10% MeOH in DCM); mp: 133–137 °C; IR ν_max_ (cm^–1^) 3278, 3073, 2933, 2220, 2110; ^1^H NMR (500 MHz, Methanol-*d*_4_) δ 1.12–1.57 (m, 13H), 1.59–1.67 (m, 1H), 1.73–1.81 (m, 1H), 1.99 (s, 3H), 3.01–3.06 (m, 2H), 3.94–4.01 (m, 2H), 4.26 (dd, *J* = 8.9, 5.4 Hz, 1H); ^13^C NMR (126 MHz, Methanol-*d*_4_) δ 21.0, 22.7, 27.4, 29.0, 29.2, 31.4, 39.7, 41.8, 53.3, 75.5, 78.5, 157.2, 170.0, 172.8; LCMS (ESI+) *m*/*z* = 306.2 [M-Boc + H]^+^; HRMS calcd for C16H27^79^BrN3O4 426.1004 [M + Na]^+^ found 426.0955.

#### (S)-5-acetamido-6-((3-bromoprop-2-yn-1-yl)amino)-6-oxohexan-1-aminium trifluoroacetate (PLK)

tert-Butyl (S)-(5-acetamido-6-((3-bromoprop-2-yn-1-yl)amino)-6-oxohexyl)carbamate (75 mg, 0.19 mmol), TFA (0.68 mL), TIPSH (60 μL), and water (60 μL) were stirred together at rt for 2 h. The reaction mixture was diluted with water and extracted with diethyl ether. The aqueous phase was evaporated to dryness to afford (S)-2-acetamido-N-(3-bromoprop-2-yn-1-yl)-6-((2,2,2-trifluoroacetyl)-λ4-azanyl)hexanamide (70 mg, 92%) as a colorless gum.

IR ν_max_ (cm^–1^) 3256, 3056, 2935, 2349, 2102, 1999, 1903; ^1^H NMR (500 MHz, Methanol-*d*_4_) δ 1.36–1.54 (m, 1H), 1.62–1.73 (m, 4H), 1.80–1.88 (m, 1H), 2.00 (s, 3H), 2.87–2.97 (m, 2H), 3.96–4.03 (m, 2H), 4.31 (dd, *J* = 8.8, 5.4 Hz, 1H); ^13^C NMR (75 MHz, Methanol-*d*_4_) δ 22.5, 23.8, 28.1, 30.5, 32.4, 40.5, 54.3, 76.9, 173.5, 173.9, C_q_ absent; LCMS (ESI+) *m*/*z* = 305.2 [M-TFA + H]^+^; HRMS calcd for C_11_H_18_^79^BrN_3_O_2_ 326.0480 [M + Na]^+^ found 326.0422.

#### (S)-2-Acetamido-N-(3-bromoprop-2-yn-1-yl)-5-(3-((2,2,5,7,8-pentamethylchroman-6-yl)sulfonyl)guanidino)pentanamide

Synthesized according to the general procedure using Ac-Arg(PMC)-OH (250 mg, 0.52 mmol). The crude product was purified by flash silica chromatography, elution gradient 0–8% MeOH in DCM, to afford (S)-2-acetamido-N-(3-bromoprop-2-yn-1-yl)-5-(3-((2,2,5,7,8-pentamethylchroman-6-yl)sulfonyl)guanidino)pentanamide (192 mg, 62%) as an off-white crystalline solid.

*R*_f_ = 0.43 (10% MeOH in DCM); mp: 112 °C (decomp.); UV λ_max_ (EtOH/nm) 251.6, 218.2; IR ν_max_ (cm^–1^) 3433, 3309, 2927, 2113, 1860; ^1^H NMR (500 MHz, Methanol-*d*_4_) δ 1.32 (s, 6H), 1.44–1.64 (m, 3H), 1.73–1.81 (m, 1H), 1.85 (t, *J* = 6.9 Hz, 2H), 1.97 (s, 3H), 2.11 (s, 3H), 2.56 (s, 3H), 2.57 (s, 3H), 2.68 (t, *J* = 6.8 Hz, 2H), 3.11–3.22 (m, 2H), 3.95 (s, 2H), 4.28 (dd, *J* = 8.7, 5.3 Hz, 1H).^13^C NMR (126 MHz, Methanol-*d*_4_) δ 10.9, 16.4, 17.5, 21.0, 21.0, 25.6, 28.9, 29.0, 32.4, 41.8, 52.9, 73.5, 75.5, 118.0, 123.6, 133.3, 134.7, 135.1, 153.3, 172.0, 172.6, C_q_ absent; LCMS (ESI+) *m*/*z* = 600.4 [M + H]^+^; HRMS calcd for C_25_H_37_^79^BrN_5_O_5_S 598.1698 [M + H]^+^ found 598.1870.

#### (S)-1-(4-Acetamido-5-((3-bromoprop-2-yn-1-yl)amino)-5-oxopentyl)guanidinium trifluoroacetate (PLR)

(S)-2-Acetamido-N-(3-bromoprop-2-yn-1-yl)-5-(3-((2,2,5,7,8-pentamethylchroman-6-yl)sulfonyl)guanidino)pentanamide (143 mg, 0.24 mmol), TFA (1.3 mL), TIPSH (0.12 mL), and water (0.12 mL) were stirred together at rt for 5 h and left to stand overnight. The reaction mixture was diluted with water and extracted with diethyl ether. The aqueous phase was evaporated to dryness. The crude product was dissolved in MeOH, stirred with activated charcoal for 1 h, and then filtered. The filtrate was evaporated to dryness to afford (S)-2-acetamido-N-(3-bromoprop-2-yn-1-yl)-5-(((2,2,2-trifluoroacetyl)- λ4-azaneyl)formimidamido)-pentanamide (38 mg, 37%) as a pale yellow gum.

IR ν_max_ (cm^–1^) 3171, 3059, 2962, 2106, 1993; ^1^H NMR (300 MHz, D_2_O) δ 1.51–1.89 (m, 4H), 2.00 (s, 3H), 3.14–3.21 (m, 2H), 3.94 (d, *J* = 2.5 Hz, 2H), 4.17–4.22 (m, 1H); ^13^C NMR (126 MHz, D_2_O) δ 21.7, 24.3, 28.1, 29.8, 40.5, 43.2, 48.9, 75.2, 156.7, 173.8, 174.4; LCMS (ESI+) *m*/*z* = 332.3 [M-TFA + H]^+^; HRMS calcd for C_11_H_18_^81^BrN_5_O_2_ 356.0521 [M + Na]^+^ found 356.0774.

#### (S)-2-Acetamido-N-(3-bromoprop-2-yn-1-yl)-3-(1-trityl-1H-imidazol-5-yl)propanamide

Synthesized according to the general procedure using Ac-His(Trt)-OH (250 mg, 0.57 mmol). The crude product was purified by flash silica chromatography, elution gradient 0–8% MeOH in DCM, to afford (S)-2-acetamido-N-(3-bromoprop-2-yn-1-yl)-3-(1-trityl-1H-imidazol-5-yl)propanamide (110 mg, 35%) as an off-white solid.

*R*_f_ = 0.40 (10% MeOH in DCM); mp: n/a, compound decomposed at 90 °C; IR ν_max_ (cm^–1^) 3264, 3057, 2923, 2343, 2222, 2118, 1908; ^1^H NMR (500 MHz, Methanol-*d*_4_) δ 1.90 (s, 3H), 2.80 (dd, *J* = 14.7, 8.9 Hz, 1H), 3.00 (dd, *J* = 14.7, 5.4 Hz, 1H), 3.86–3.99 (m, 2H), 4.53–4.61 (m, 1H), 6.72 (s, 1H), 7.10–7.18 (m, 6H), 7.35–7.43 (m, 10H); ^13^C NMR (126 MHz, Methanol-*d*_4_) δ 22.6, 30.5, 31.6, 54.6, 76.8, 76.9, 121.1, 129.3, 129.3, 130.9, 137.6, 139.4, 143.6, 173.0, 173.2, C_q_ absent; LCMS (ESI+) *m*/*z* = 557.4 [M + H]^+^; HRMS calcd for C_30_H_28_^79^BrN_4_O_2_ 555.1395 [M + H]^+^ found 555.1508.

#### (S)-5-(2-Acetamido-3-((3-bromoprop-2-yn-1-yl)amino)-3-oxopropyl)-1H-imidazol-3-ium trifluoroacetate (PLH)

(S)-2-Acetamido-N-(3-bromoprop-2-yn-1-yl)-3-(1-trityl-1H-imidazol-5-yl)propanamide (67 mg, 0.12 mmol), TFA (0.6 mL), TIPSH (55 μL), and water (55 μL) were stirred together at rt for 3 h. The reaction mixture was diluted with water and extracted with diethyl ether. The aqueous phase was evaporated to dryness. The crude product was redissolved in water, extracted again with diethyl ether, and evaporated to afford (S)-2-acetamido-N-(3-bromoprop-2-yn-1-yl)-3-(1-(2,2,2-trifluoroacetyl)-1H- λ4-imidazol-5-yl)propanamide (24 mg, 49%) as a pale yellow gum, which crystallized on standing.

IR ν_max_ (cm^–1^) 3224, 3049, 2842, 2627, 2119, 1927; ^1^H NMR (500 MHz, Methanol-*d*_4_) δ 1.97 (s, 3H), 3.04 (dd, *J* = 15.3, 8.1 Hz), 3.26 (dd, *J* = 15.3, 6.0 Hz, 1H), 3.94–4.03 (m, 2H), 4.70 (dd, *J* = 8.1, 6.0 Hz, 1H), 7.31 (s, 1H), 8.80 (s, 1H); ^13^C NMR (126 MHz, Methanol-*d*_4_) δ 21.1, 26.7, 29.2, 41.9, 50.0, 75.4, 117.0, 129.8, 133.6, 170.4, 172.0; LCMS (ESI+) *m*/*z* = 314.0 [M-TFA + H]^+^; HRMS calcd for C_11_H_13_^79^BrN_4_O_2_ 335.0120 [M + Na]^+^ found 335.0239.

#### (S)-N,N′-(6-((3-Bromoprop-2-yn-1-yl)amino)-6-oxohexane-1,5-diyl)diacetamide (PLKAc)

Synthesized according to the general procedure using Nα,ε-bis-acetyl-l-lysine (304 mg, 1.3 mmol) and evaporating the reaction mixture to afford the crude product without aqueous work-up. The crude product was purified twice by flash silica chromatography, elution gradients 0–15% MeOH in DCM and 0–15% MeOH in EtOAc, to afford (S)-N,N′-(6-((3-bromoprop-2-yn-1-yl)amino)-6-oxohexane-1,5-diyl)diacetamide (28 mg, 9%) as a white solid.

*R*_f_ = 0.32 (15% MeOH in EtOAc); mp: 167 °C (decomp.); IR ν_max_ (cm^–1^) 3276, 3085, 2924, 2858, 2222, 2118; ^1^H NMR (500 MHz, Methanol-*d*_4_) δ 1.29–1.45 (m, 2H), 1.48–1.55 (m, 2H), 1.60–1.69 (m, 1H), 1.74–1.82 (m, 1H), 1.93 (s, 3H), 1.99 (s, 3H), 3.16 (t, *J* = 7.0 Hz, 2H), 3.98 (d, *J* = 1.8 Hz, 2H), 4.26 (dd, *J* = 8.9, 5.4 Hz, 1H); ^13^C NMR (126 MHz, Methanol-*d*_4_) δ 22.4, 22.6, 24.2, 30.0, 30.4, 32.7, 40.2, 43.2, 54.6, 76.9, 173.2, 173.4, 174.2; LCMS (ESI+) *m*/*z* = 368.2 [M + Na]^+^; HRMS calcd for C_13_H_21_^79^BrN_3_O_3_ 346.0766 [M + H]^+^ found 346.0776.

#### 4-Chloro-1-methyl-3-nitropyridin-2(1H)-one

4-Chloro-3-nitropyridin-2(1H)-one (1 g, 5.73 mmol), iodomethane (392 μL, 1.1 equiv), cesium carbonate (2.24 g, 1.2 equiv), and DMF (15 mL) were combined and heated to 80 °C under microwave irradiation for 45 min. The mixture was allowed to cool to r.t., diluted with diethyl ether, filtered, and the solvent was removed in vacuo. The residue was purified by flash column chromatography on silica (24 g, 50–80% EtOAc/petrol) to give a yellow solid (955 mg, 89%).

*R*_f_ 0.60 (5% MeOH/DCM); ^1^H NMR (500 MHz; CDCl_3_) δ 3.63 (s, 3H), 6.32 (d, *J* = 7.4 Hz, 1H), 7.39 (d, *J* = 7.4 Hz, 1H).

#### 4-((2-Hydroxyethyl)(methyl)amino)-1-methyl-3-nitropyridin-2(1H)-one

2-(Methylamino)ethanol (64 μL, 3 equiv) was added to a mixture of 4-chloro-1-methyl-3-nitropyridin-2(1H)-one (50 mg, 0.266 mmol) in MeOH (0.5 mL), and the mixture was heated to 120 °C under microwave irradiation for 1 h. The solvent was removed in vacuo, and the mixture was partitioned between EtOAc (20 mL) and NaHCO_3_ (10% aq., 10 mL). Saturated aqueous NaCl (10 mL) was added to the aqueous layer and extracted with CH2Cl2 (7 × 20 mL). The organic extracts were combined, dried (MgSO_4_), and the solvent was removed in vacuo to give a yellow solid (60 mg, 100%).

*R*_f_ 0.35 (5% MeOH/DCM); ^1^H NMR (500 MHz; CDCl_3_) δ 2.96 (s, 3H), 3.47 (s, 3H), 3.53 (t, *J* = 5.3 Hz, 2H), 3.85 (t, *J* = 5.3 Hz, 2H), 6.04 (d, *J* = 8.0 Hz, 1H), 7.12 (d, *J* = 8.0 Hz, 1H); LCMS (ESI+) 228.1 [M + H]^+^.

#### 2-(Methyl(1-methyl-3-nitro-2-oxo-1,2-dihydropyridin-4-yl)amino)ethyl methanesulfonate

Methanesulfonyl chloride (30 μL, 0.40 mmol, 1.5 equiv) was added to 4-((2-hydroxyethyl)(methyl)amino)-1-methyl-3-nitropyridin-2(1H)-one (60 mg, 0.26 mmol) and Et_3_N (74 μL, 2 equiv) in DCM (1 mL) at 0 °C, and the mixture was stirred at 0 °C for 1.5 h. Further, methanesulfonyl chloride (10 μL, 0.5 equiv) was added and the mixture was stirred at r.t. for 1 h. The mixture was partitioned between DCM (3 × 20 mL) and water (10 mL). The organic extracts were combined, dried (MgSO_4_), and the solvent was removed in vacuo to give the title compound as a yellow gum (76 mg, 94%).

*R*_f_ 0.45 (5% MeOH/DCM); ^1^H NMR (500 MHz; CDCl_3_) δ 3.02 (s, 3H), 3.04 (s, 3H), 3.49 (s, 3H), 3.68 (t, *J* = 5.5 Hz, 2H), 4.36 (t, *J* = 5.5 Hz, 2H), 5.94 (d, *J* = 7.9 Hz, 1H), 7.17 (d, *J* = 7.9 Hz, 1H); LCMS (ESI+) 306.1 [M + H]^+^.

#### 1,6-Dimethyl-2,3,4,6-tetrahydropyrido[3,4-*b*]pyrazin-5(1H)-one (**8**)

2-(Methyl(1-methyl-3-nitro-2-oxo-1,2-dihydropyridin-4-yl)amino)ethyl methanesulfonate (76 mg, 0.25 mmol) was dissolved in MeOH (5 mL) and hydrogenated on a ThalesNano H-cube (10% Pd/C, 1 mL/min, 50 °C, full H_2_ mode, continuous recycling of reaction mixture) for 3 h. The solvent was removed in vacuo, and the mixture was partitioned between DCM (3 × 20 mL) and NaHCO_3_ (10% aq., 10 mL). The organic extracts were combined, dried (MgSO_4_), and the solvent was removed in vacuo. The residue was purified by flash column chromatography on silica (4 g, 0–5% MeOH/DCM) to give a black gum (16 mg, 36%).

*R*_f_ 0.25 (5% MeOH/DCM); ^1^H NMR (500 MHz; CDCl3) δ 2.90 (s, 3H), 3.33–3.37 (m, 2H), 3.37–3.42 (m, 2H), 3.50 (s, CONMe, 3H), 4.27 (br s, 1H), 5.85 (d, *J* = 7.5 Hz, 1H), 6.68 (d, *J* = 7.5 Hz, 1H); ^13^C NMR (125 MHz; CDCl3) δ 36.7, 38.0, 39.8, 49.8, 96.3, 118.0, 126.7, 137.4, 156.2; LCMS (ESI+) 180.1 [M + H]^+^.

#### 3-Amino-1-methylpyridin-2(1H)-one

1-Methyl-3-nitropyridin-2(1H)-one (74 mg, 0.48 mmol) was dissolved in MeOH (2.4 mL) and hydrogenated on a ThalesNano H-cube (10% Pd/C, 1 mL/min, 50 °C, Full H_2_ mode, continuous recycling of reaction mixture) until the reaction was complete. The solvent was removed in vacuo to give the product as a dark brown oil (60 mg, 99%).

*R*_f_ 0.74 (NH silica; 10% MeOH in DCM); UV λ_max_ (EtOH/nm) 309.6, 258.6; IR ν_max_ (cm^–1^) 2653, 1645 (C=O), 1592, 1516; ^1^H NMR (500 MHz, DMSO-*d*_6_) δ 3.43 (s, 3H), 5.06 (br s, 2H), 6.01 (dd, *J* = 6.8, 7.1 Hz, 1H), 6.42 (dd, *J* = 1.8, 7.1 Hz 1H), 6.88 (dd, *J* = 1.8, 6.8 Hz, 1H); ^13^C NMR (125 MHz, DMSO-*d*_6_) δ 37.1, 106.5, 110.4, 125.5, 138.7, C_q_ absent; LCMS (ESI+) *m*/*z* 125.0 [M + H]^+^; HRMS calcd for C_6_H_9_N_2_O_1_ 25.0715 [M + H]^+^ found 125.0711.

#### tert-Butyl 4-formylpiperidine-1-carboxylate

Dess-Martin periodinane (382 mg, 0.90 mmol), tert-butyl 4-(hydroxymethyl)piperidine-1-carboxylate (155 mg, 0.72 mmol), and DCM (2.4 mL) were combined and stirred at r.t. for 18 h. The mixture was diluted with DCM (9 mL) and sat. aq. Na_2_S_2_O_3_ (9 mL), the organics were extracted with DCM (3 × 10 mL/mmol) and EtOAc (2 × 10 mL/mmol), and dried (Na_2_SO_4_). The crude product was purified by flash chromatography (silica; 0–50% EtOAc/petrol) to give the product as a colorless oil (82 mg, 53%).

*R*_f_ 0.61 (50% EtOAc/petrol, KMnO4 stain); UV λ_max_ (EtOH/nm) 287.0, 218.8; IR ν_max_ (cm^–1^) 2929, 2856, 2713, 1705 (C=O), 1685 (C=O); ^1^H NMR (500 MHz, CDCl_3_) δ 1.45 (s, 9H), 1.48–1.58 (m, 2H), 1.87–1.89 (m, 2H), 2.38–2.42 (m, 1H), 2.90–2.95 (m, 2H), 3.98 (br s, 2H), 9.66 (s, 1H); ^13^C NMR (125 MHz, CDCl_3_) δ 25.2, 28.4, 48.0, 79.8, 154.7, 203.0, one CH absent; LCMS (ESI+) mass ion not detected.

#### tert-Butyl 4-(((1-methyl-2-oxo-1,2-dihydropyridin-3-yl)amino)methyl)piperidine-1-carboxylate

3-Amino-1-methylpyridin-2(1H)-one (42 mg, 0.34 mmol), tert-butyl 4-formylpiperidine-1-carboxylate (72 mg, 0.34 mmol), DCM (1.4 mL), and NaBH(OAc)_3_ (108 mg, 0.51 mmol) were combined and stirred at r.t. for 18 h. The mixture was diluted with DCM (5 mL), washed with water (5 mL), and further extracted with DCM (3 × 5 mL). Solid NaHCO_3_ was added to the aqueous layer and further extracted with EtOAc (3 × 5 mL). The combined organic extracts were dried (Na_2_SO_4_). The crude product was purified by flash chromatography (silica; 0–6% MeOH in DCM) to give the product as a green oil (71 mg, 65%).

*R*_f_ 0.37 (10% MeOH in DCM); UV λ_max_ (EtOH/nm) 322.3, 263.0; IR ν_max_ (cm^–1^) 3341 (NH), 2972, 2929, 2846, 1684 (C=O), 1636 (C=O), 1594; ^1^H NMR (500 MHz, CDCl_3_) δ 1.13–1.25 (m, 2H), 1.46 (s, 9H), 1.73–1.78 (m, 3H), 2.66–2.71 (m, 2H), 2.97 (d, *J* = 6.4 Hz, 2H), 3.56 (s, 3H), 4.13 (br s, 2H), 6.12 (dd, *J* = 6.7, 7.4 Hz, 1H), 6.16 (dd, *J* = 1.7 and 7.4 Hz, 1H), 6.61 (dd, *J* = 1.7, 6.7 Hz, 1H), NH absent; ^13^C NMR (125 MHz, CDCl_3_) δ 28.5, 29.7, 30.2, 35.8, 37.4, 49.0, 79.4, 106.0, 107.1, 123.3, C_q_ absent; LCMS (ESI+) *m*/*z* 322.3 [M + H]^+^; HRMS calcd for C_12_H_20_ON_3_ 222.1601 [M-COOC(CH_3_)_3_ + H]^+^ found 222.1602.

#### 1-Methyl-3-((piperidin-4-ylmethyl)amino)pyridin-2(1H)-one dihydrochloride (**9**)

HCl (4 M in dioxane, 0.3 mL, 1.17 mmol), tert-butyl 4-(((1-methyl-2-oxo-1,2-dihydropyridin-3-yl)amino)methyl)piperidine-1-carboxylate (54 mg, 0.17 mmol), and dioxane (0.3 mL) were stirred at r.t. for 16 h. The mixture was concentrated in vacuo. The residue was dissolved in DCM 10 mL, washed with sat. aq. NaHCO_3_ (10 mL), and dried over Na_2_SO_4_ to give the product as a pale blue hygroscopic solid (22 mg, 58%).

*R*_f_ 0.16 (10% MeOH and 10% AcOH in DCM); UV λ_max_ (EtOH/nm) 315.8, 264.4, 207.0; IR ν_max_/cm^–1^ 3351 (NH), 3020, 2908, 2792, 2709, 2634, 2564, 2490, 2454, 2390, 1661 (C=O), 1561; ^1^H NMR (500 MHz, CDCl_3_) δ 1.30–1.37 (2H, m), 1.80–1.88 (3H, m), 2.80–2.82 (2H, m), 2.96 (2H, d, *J* = 6.5 Hz), 2.24–2.27 (2H, m), 3.44 (3H, s), 6.10 (1H, dd, *J* = 6.9 and 6.9 Hz), 6.23 (1H, dd, *J* = 1.5 and 6.9 Hz), 6.89 (1H, dd, *J* = 1.5 and 6.9 Hz), 8.45 (1H, br s), 8.77 (1H, br s); ^13^C NMR (125 MHz, CDCl3) δC 26.9, 32.6, 37.1, 43.3, 47.9, 106.0, 106.6, 124.7, 138.3, 157.6; MS (ESI+) *m*/*z* 222.3 [M + H]^+^; HRMS calcd for C_12_H_20_ON_3_ 222.1601 [M + H]^+^ found 222.1599.

#### 1-Methyl-3-nitro-4-((pyridin-2-ylmethyl)amino)pyridin-2(1H)-one

2-Picolylamine (27 μL, 1 equiv) was added to 4-chloro-1-methyl-3-nitropyridin-2(1H)-one (50 mg, 0.266 mmol) and Et_3_N (41 μL, 1 equiv) in DCM (1 mL), and the mixture was heated to 50 °C under microwave irradiation for 30 min. The mixture was partitioned between DCM (3 × 10 mL) and NaHCO_3_ (10% aq., 10 mL). The organic extracts were combined, dried (MgSO_4_), and the solvent was removed in vacuo. The residue was purified by flash column chromatography on silica (4 g, 0–8% MeOH/DCM) to give the title compound (52 mg, 75%).

*R*_f_ 0.45 (8% MeOH/DCM); ^1^H NMR (500 MHz; CDCl_3_) δ 3.47 (s, 3H), 4.67 (d, *J* = 5.4 Hz, 2H), 5.86 (d, *J* = 7.9 Hz, 1H), 7.23 (d, *J* = 7.9 Hz, 1H), 7.25–7.30 (m, 2H), 7.18 (td, *J* = 7.6, 1.7 Hz, 1H), 8.63 (d, *J* = 5.0 Hz, 1H), 9.83–9.94 (m, 1H).

#### 3-Amino-1-methyl-4-((pyridin-2-ylmethyl)amino)pyridin-2(1H)-one (**10**)

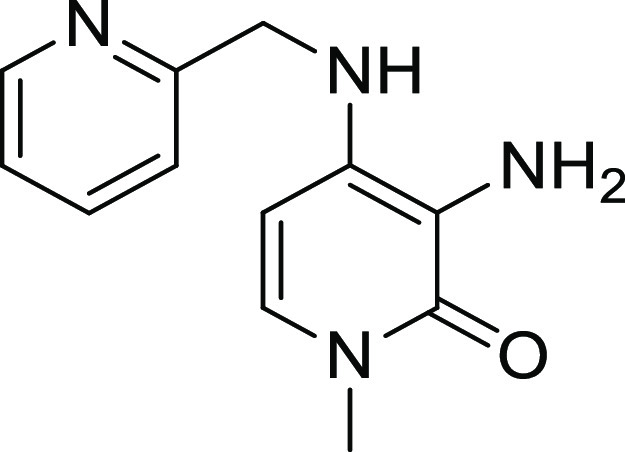



1-Methyl-3-nitro-4-((pyridin-2-ylmethyl)amino)pyridin-2(1H)-one (50 mg, 0.19 mmol) was dissolved in MeOH (2 mL) and hydrogenated on a ThalesNano H-cube (10% Pd/C, 1 mL/min, 50 °C, Full H_2_ mode, continuous recycling of reaction mixture) for 2 h. The solvent was removed in vacuo, and the residue was purified by flash column chromatography on silica (4 g, 1–12% MeOH/DCM) to give a beige solid (13 mg, 30%).

*R*_f_ 0.3 (8% MeOH/DCM); ^1^H NMR (500 MHz; CDCl_3_) δ 3.52 (s, 3H), 4.52 (s, 2H), 5.04 (br s, 1H), 5.85 (d, *J* = 7.5 Hz, 1H), 6.82 (d, *J* = 7.5 Hz, 1H), 7.21 (dd, *J* = 4.9, 7.4 Hz, 1H), 7.28 (d, *J* = 7.8 Hz, 1H), 7.67 (td, *J* = 7.8, 1.7 Hz, 1H), 8.58 (d, *J* = 4.9 Hz, 1H); MS (ESI+) 231.1 [M + H]^+^.

## Supporting Information Available

X-ray structures have been deposited in the PDB. Accession numbers are given in Tables S1 and S2. The authors will release the atomic coordinates upon article publication (CSV)
Summary tables of the FragLite and PepLite binding events, NMR stability studies, methods for protein expression and crystallization and analytical data for synthesized compounds, and molecular formula strings table (PDF)

## Abbreviations Used

ATAD2: ATPase family AAA domain containing 2
BRD4: bromodomain-containing 4
CDK2: cyclin-dependent kinase 2
DSI: diamond, SGC, and iNEXT
DTT: dithiothreitol
HTRF: homogeneous time-resolved fluorescence
PanDDA: pan-dataset density analysis
TCEP: (tris(2-carboxyethyl)phosphine)
TFA: trifluoroacetic acid
