# Supplementary material for: Mapping Ligand Interactions of Bromodomains BRD4 and ATAD2 with FragLites and PepLites—Halogenated Probes of Druglike and Peptide-like Molecular Interactions
Source: J Med Chem. 2022 Nov 11;65(22):15416–32. doi: 10.1021/acs.jmedchem.2c01357 (PMC9706561; doi:10.1021/acs.jmedchem.2c01357)

Supporting information

Mapping ligand interactions of bromodomains BRD4 and ATAD2 with FragLites and PepLites – halogenated probes of drug-like and peptide-like molecular interactions

Gemma Davison,[†§] Mathew P. Martin,[†§] Shannon Turberville,[‡§] Selma Dormen,[†] Richard Heath,[‡] Amy B. Heptinstall,[†] Marie Lawson,[†] Duncan C. Miller,[†] Yi Min Ng,[‡] James N. Sanderson,[†] Ian Hope,[‡] Daniel J. Wood,[‡] Céline Cano,[†] Jane A. Endicott,[‡] Ian R. Hardcastle,[†] Martin E. M. Noble,[‡]* Michael J. Waring.[†]*

[†]Cancer Research Horizons Therapeutic Innovation, Newcastle Drug Discovery Unit, Newcastle University Centre for Cancer, Chemistry, School of Natural and Environmental Sciences, Newcastle University, Bedson Building, Newcastle upon Tyne, NE1 7RU, UK.

[‡]Cancer Research Horizons Therapeutic Innovation, Newcastle Drug Discovery Unit, Newcastle University Centre for Cancer, Newcastle University, Paul O'Gorman Building, Framlington Place, Newcastle upon Tyne, NE2 4AD, UK.

Contents

Supplementary Tables 2

Supplementary Figures..2

Protein Expression and Crystallisation 3

Analytical data 4

Supplementary Tables

Table S1. Summary of binding events for FragLite Screening of ATAD2 and BRD4

ATAD2 Contacts within 4Å for Lipophilic Interactions /lattice contact /halogen Interaction & within 3.2Å for Hydrogen bonding

FragLite	PDB	Status	Site	Ortho/Allosteric	Hydrogen bonding	Lipophilic Interaction	Lattice contacts	Halogen Interctions
1	7QUK	PanDDA	Site 4	Allosteric	yes	yes	yes	no
2	7QUM	PanDDA	Site 4	Allosteric	yes	yes	yes	no
3	7PPX	Anomalous	Site 3	Allosteric	yes	yes	no	yes
6	7QWO	PanDDA	Site 4	Allosteric	yes	yes	yes	no
7	7QX1	PanDDA	Site 1 and 2	Orthosteric/Allosteric	yes (1) yes (2)	yes (1) yes (2)	no (1) yes (2)	yes (1) no (2)
10	7QXT	Anomalous	Site 2	Allosteric	no	yes	yes	yes
16	7QU7	Anomalous	Site 1	Orthosteric	yes	yes	no	no
18	7QYK	Anomalous	Site 3	Allosteric	yes	yes	no	yes
23	7QYL	Anomalous	Site 6	Allosteric	no	yes	no	yes
28	7QZM	PanDDA	Site 5	Allosteric	no	yes	yes	no
29	7QZY	PanDDA	Site 6 (two copies)	Allosteric	yes	yes	yes	no
31	7QZZ	PanDDA	Site 7 and 5	Allosteric	yes (7) yes (5)	yes (7) yes (5)	no (7) no (5)	yes (7) yes (5)
33	7R00	Anomalous	Site 1	Orthosteric	yes	yes	no	no

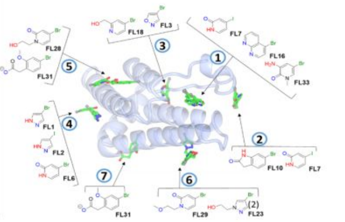

BRD4

FragLite	PDB	Status	Site	Ortho/Allosteric	Hydrogen bonding	Lipophilic Interaction	Lattice Contacts	Halogen Interactions
1	7Z9W	Anomalous	Site 2	Allosteric	yes	yes	no	yes
2	7Z9Y	Anomalous	Site 2 & 4	Allosteric	no (2) yes (4)	yes (2) yes (4)	yes (2) no (4)	yes (2) yes (4)
4	7ZA6	PanDDA	Site 2	Allosteric	no	yes	yes	yes
5	7ZA7	Anomalous	Site 1 & 2	Orthosteric/Allosteric	yes (1) no (2)	yes (1) yes (2)	yes (1) yes (2)	yes (1) yes (3)
6	7ZA8	Anomalous	Site 1	Orthosteric	yes	yes	no	yes
7	7ZA9	Anomalous	Site 5	Allosteric	yes	yes	yes	yes
10	7ZE6	Anomalous	Site 1	Orthosteric	yes	yes	yes	yes
11	7ZAA	Anomalous	Site 1	Orthosteric	yes	yes	no	yes
12	7ZAD	Anomalous	Site 1	Orthosteric	yes	yes	yes	yes
15	7ZAE	PanDDA	Site 1	Orthosteric	yes	yes	yes	yes
16	7ZAJ	Anomalous	Site 1	Orthosteric	yes	yes	no	yes
18	7ZAR	Anomalous	Site 1	Orthosteric	yes	yes	no	yes
19	7ZAQ	Anomalous	Site 1	Orthosteric	yes	yes	yes	yes
20	7ZAT	Anomalous	Site 1	Orthosteric	yes	yes	no	yes
21	7ZE7	Anomalous	Site 1 & 3	Orthosteric/Allosteric	yes (1) no (3)	yes (1) yes (3)	yes (1) no (3)	yes (1) yes (3)
22	7ZEF	Anomalous	Site 1	Orthosteric	yes	yes	yes	yes
23	7ZEN	PanDDA	Site 2	Allosteric	yes	yes	no	yes
24	7ZFN	PanDDA	Site 1 & 3	Orthosteric/Allosteric	yes (1) no (3)	yes (1) yes (3)	yes (1) no (3)	yes (1) yes (3)
28	7ZFO	Anomalous	Site 1	Orthosteric	yes	yes	no	yes
29	7ZFQ	Anomalous	Site 1	Orthosteric	yes	yes	no	yes
32	7ZFS	Anomalous	Site 1	Orthosteric	yes	yes	no	yes
33	7ZFT	Anomalous	Site 1	Orthosteric	yes	yes	no	yes

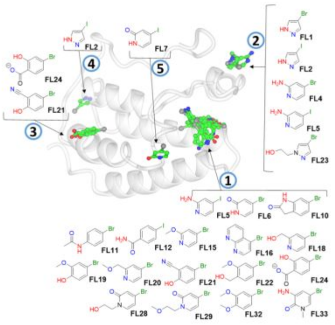

Table S2. Summary of binding events for PepLite Screening of ATAD2 and BRD4

ATAD2 Contacts within 4Å for Lipophilic Interactions /lattice contact /halogen Interaction & within 3.2Å for Hydrogen bonding

PepLite	NCL	Status	Site	Ortho/Allosteric
Isoleucine	7R05	Anomalous	Site 1	Orthosteric
Glutamine	7R0Y	PanDDA	Site 6	Allosteric
Asparagine	7Z9H	PanDDA	Site 1	Orthosteric
Alanine	7Z9I	Anomalous	Site 1	Orthosteric
Glycine	7Z9J	Anomalous	Site 6	Allosteric
Valine	7Z9N	Anomalous	Site 1	Orthosteric
Tyrosine	7Z9O	PanDDA	Site 6	Allosteric
Arginine	7Z9S	PanDDA	Site 6	Allosteric
Ace-lys	7Z9U	Anomalous	Site 1	Orthosteric

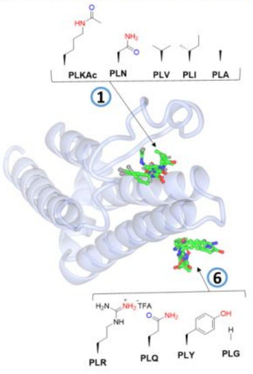

BRD4

PepLite	NCL	Status	Site	Ortho/Allosteric
Proline	7ZFU	Anomalous	Site 1	Orthosteric
Alanine	7ZFV	Anomalous	Site 1	Orthosteric
Glycine	7ZFY	Anomalous	Site 1	Orthosteric
Valine	7ZFZ	Anomalous	Site 1	Orthosteric
Tyrosine	7ZG1	Anomalous	Site 1	Orthosteric
Ace-lys	7ZG2	Anomalous	Site 1	Orthosteric

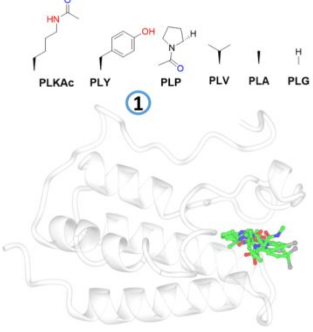

Supplementary Figures

Figure S1. NMR studies on stability of PLE over 48 hours in pH 7.4 phosphate buffer with a) no reducing agent; b) DTT; c) TCEP

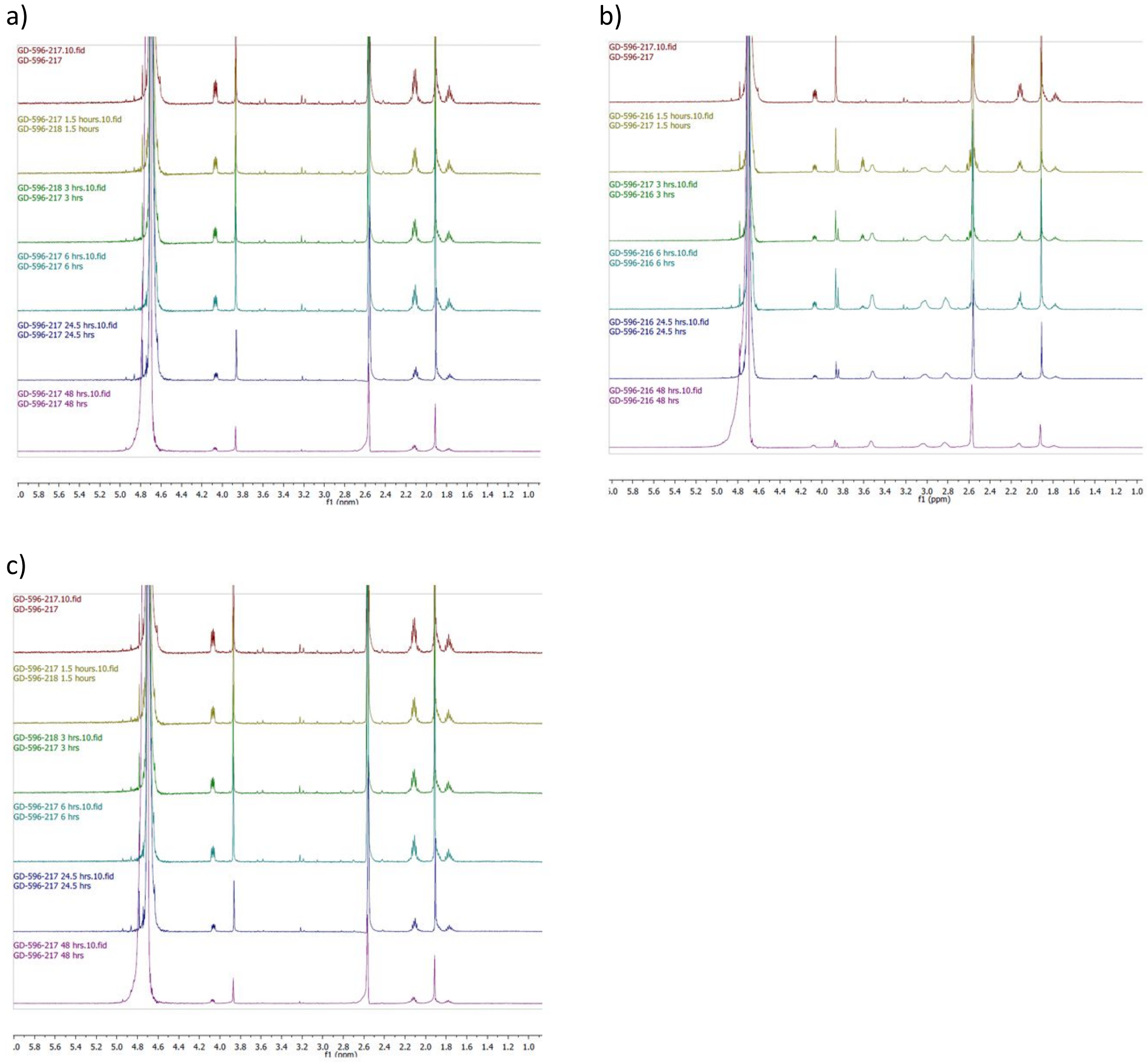

Protein Expression and Crystallisation

Expression

Recombinant human ATAD2 (res. 981-1108) and BRD4 (res. 40-168) were cloned into a pET 28b-pET28b-^{6}His vector (Astex Pharmaceuticals). pET28b-^{6}His BRD4 (Astex Pharmaceuticals) or pET28b-^{6}His ATAD2 (Astex Pharmaceuticals) were transformed and expressed in Rosetta BL21 (DE3) pLyS *E.coli* (Novagen) using the standard protocol. The transformant was on kanamycin (50 µg mL^{-1}) containing agar plates (37 °C, 16 hours). The cells were cultured at 37 °C, 200 rpm in auto-induction medium, containing kanamycin (50 µg mL^{-1}), until optical density was reached (OD_{600nm} = 0.6) whereby the temperature was lowered to 20 °C for further incubation (16 hours). The cell culture was harvested by centrifugation (4000 xg, 20 minutes, 4 °C). The resulting pellet was flash frozen in dry ice and stored at -20 °C.

Purification

Pellet was thawed and resuspended in lysis buffer (50mM HEPES pH 7.5 or 8.5 (for BRD4 and ATAD2 respectively), 500 mM NaCl, 30 mM Imidazole, 10 mM $MgCl_2$, 0.5 mM TCEP plus 25 µg mL^{-1} Lysozyme, 2 µg mL^{-1} DNase I, 10 µg mL^{-1} RNase). The cell suspension was lysed using sonication (5 minutes, pulsed for 20 seconds on and 40 seconds off, 30% amplitude) and the homogenate was centrifuged (20,000 xg, 1 hour, 4 °C). The supernatant was recovered and filtered (0.2 µM) for separation using metal immobilised Ni affinity chromatography.

The lysate was loaded on a 5ml HisTrap FF (GE Healthcare), which had been pre equilibrated with 50mM HEPES pH 7.5 or 8.5 (for BRD4 and ATAD2 respectively), 500 mM NaCl, 30 mM Imidazole, 0.5 mM TCEP, using an ÄKTA Pure chromatography system . The target protein peak fractions were pooled following the gradient of imidazole from 30mM to 250mM. The target protein was incubated with Thrombin (Sigma-Aldrich) at 4 °C whilst dialysing into
10 mM HEPES pH 7.5 or 8.5 (for BRD4 and ATAD2 respectively), 150 mM NaCl, 0.5 mM TCEP) using dialysis tubing (SnakeSkin 3.5K MWCO, ThermoFisher scientific) over 16 hours. The untagged protein was concentrated to ~5 mg mL^{-1} using an ultra-15 3000 filter (Amicon) at 4 °C. The concentrated protein was further purified by size exclusion chromatography using HiLoad 26/600 Superdex 200pg column (BRD4) or HiLoad 16/600 superdex 75pg colum (ATAD2) pre-equilibrated with their respective dialysis buffers. Target protein containing fractions were collected and concentrated to 5-10 mg mL^{-1}. Aliquots were flash frozen and stored at -80 °C for crystallisation.

Crystallisation
Crystal trials were initiated at 20 °C with a coarse screen of Morpheus (Molecular dimensions) for BRD4. For BRD4 drops were set up at 1:1 or 2:1 protein:well solution ratio (300 nl) using a Mosquito robot (TTP Labtech) with protein solution concentration of ~9 mg mL^{-1}. Crystals were identified in 0.1 M Buffer system 3 (1 M Tris, 1 M BICINE, pH 8.5), 30 % Precipitant solution 3 (20% PEG 4000, 40%) and 90 mM Halogens Mix (NaF, NaBr and NaI) solution. The crystallisation conditions were then optimised using this condition to 0.1 M Buffer system 3 pH 8.5, 34-44 % Precipitant mix 3 and 60-80 mM Halogens, with drops 1:1 or 2:1 protein:well solution ratio (300nl).
For ATAD2 drops were set up as described for BRD4 but the AmSO4 suite (Qiagen) screen was used instead. Single crystals were observed with 0.1M HEPES pH 7.0, 2.4 $AmSO_4$ with protein concentration solution of ~5 mg mL^{-1}. Conditions were optimised in plates set up manually where protein was mixed 1:1 with precipitant solution in 2 µL drops. Optimal conditions were 0.1M Bis-tris pH 5.5, 2.0-2.3 $AmSO_4$. In both cases crystals were visible in 2-3 days.
BRD4 and ATAD2 crystals were soaked for 24 hours in 50 mM compound in 10% ethylene glycol in their respective crystallisation solution. Crystallisation conditions yielded cryo protection for BRD4, whereas ATAD2 crystals were harvested in 30 % ethylene glycol prepared in ATAD2 crystallisation solution. Both BRD4 and ATAD2 were flash cooled in liquid N_2 before data collection.

Data collection and analysis
X-ray diffraction data were collected at - 200° at Diamond Light Source, Oxford, UK.
Collected data was processed in DIALS. The structures were solved using molecular replacement (DIMPLE and isomorphous ligand replacement) and using the PDB codes 3DAI and 4LYI, as search models. Refinement was carried out a combination of REFMAC5 and manual model building on COOT. The FragLite restraints and descriptions were generated using AceDRG.
Figures were prepared using the CCP4 molecular graphics programme CCP4mg50.
PanDDA30 was undertaken using 33 FragLite datasets against a variance model derived from 42 apo CDK2 crystal datasets.
Derived PanDDA maps were analyzed using the panda.inspect command and results tabulated appropriately.

Analytical data
Cmpd 8: ^{1}H NMR ($CDCl_3$)

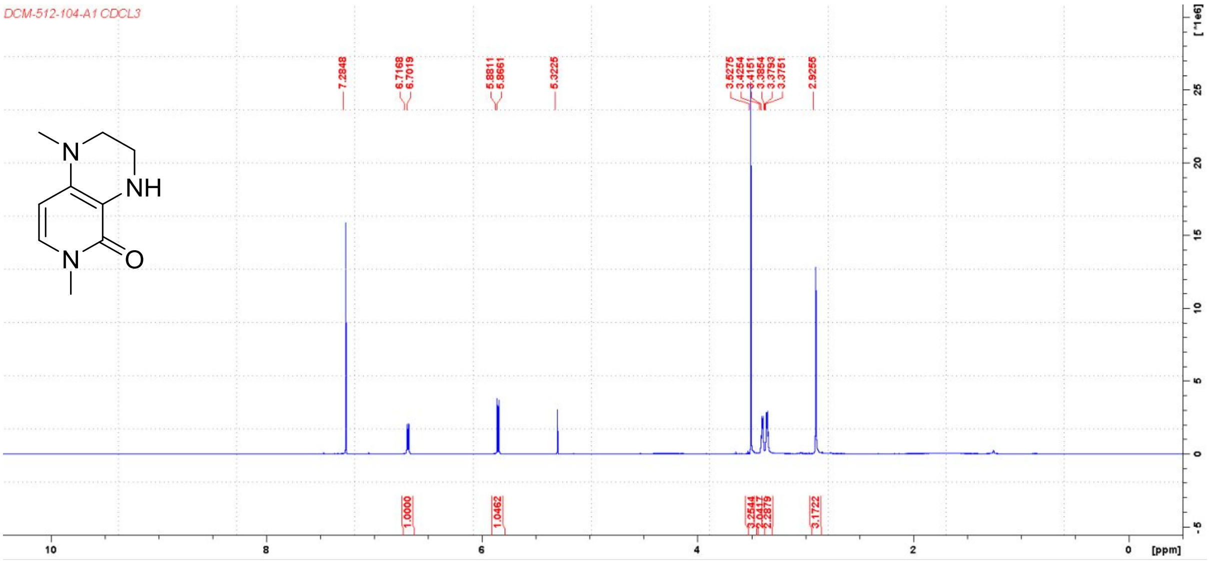

Cmpd 8: ^{13}C NMR ($CDCl_3$)

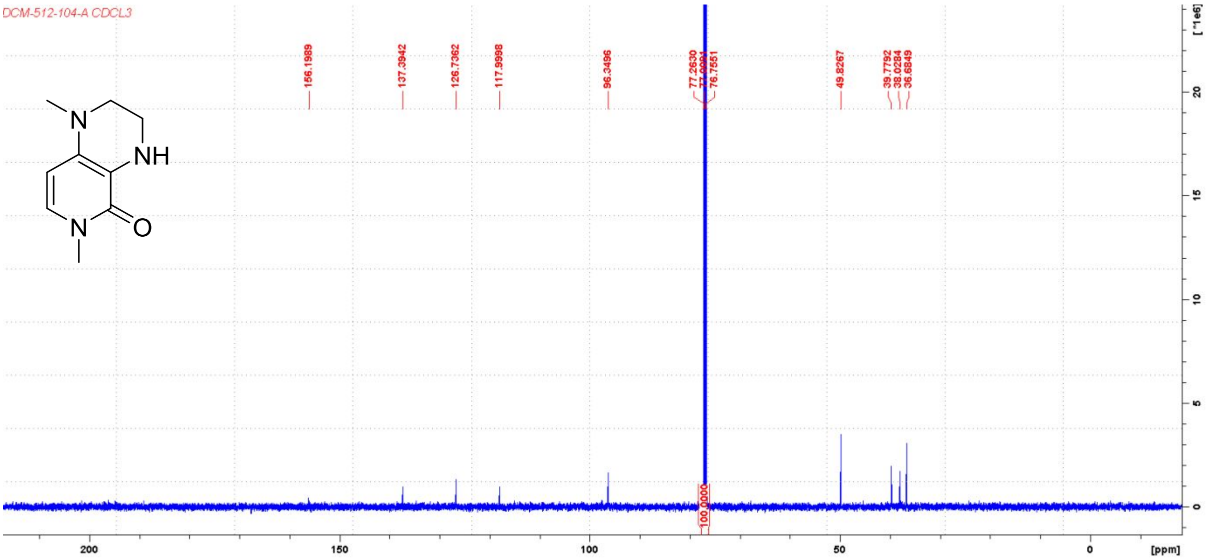

Cmpd 9: ^{1}H NMR ($CDCl_3$)

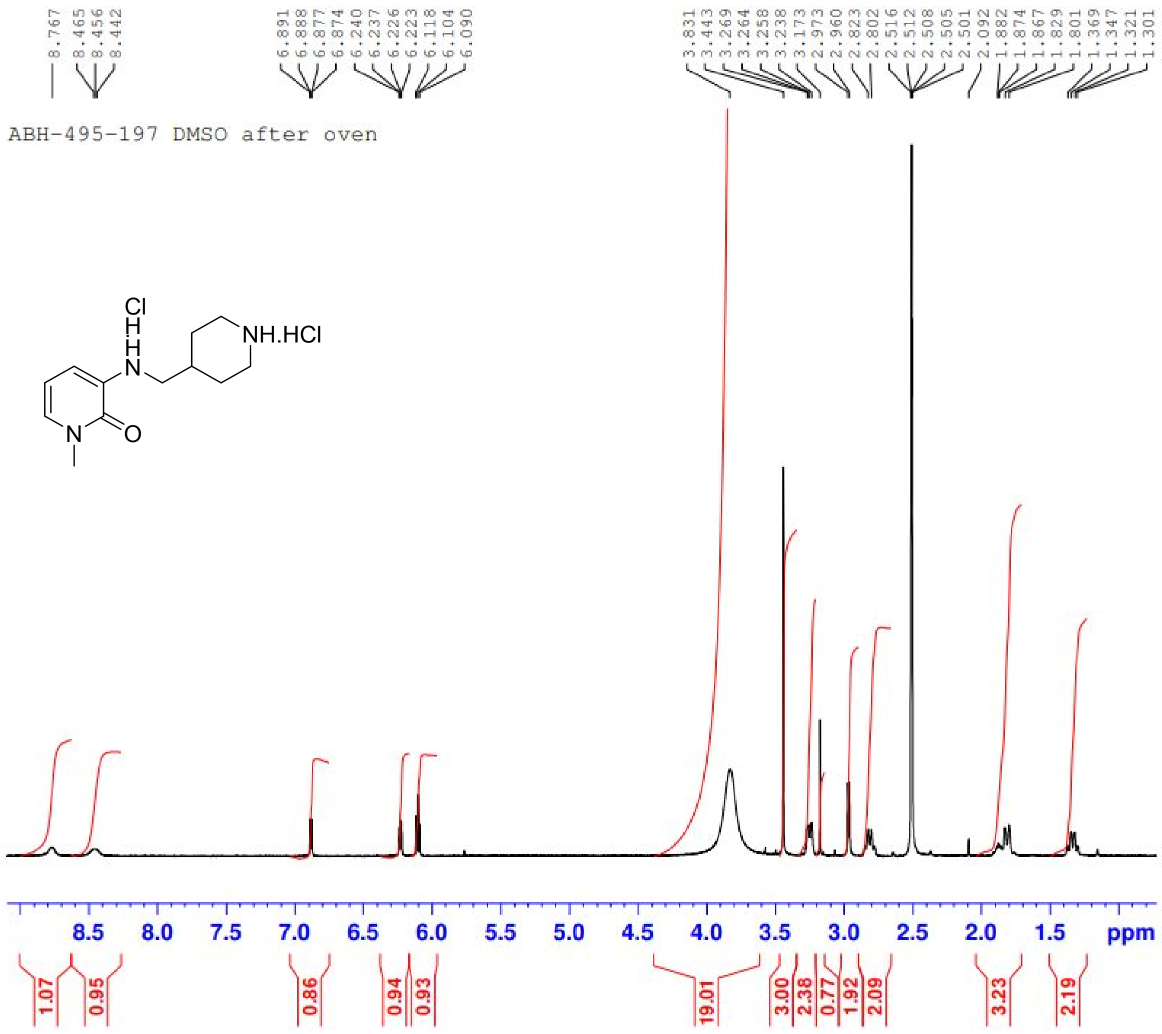

Cmpd 9: ^{13}C NMR ($CDCl_3$)

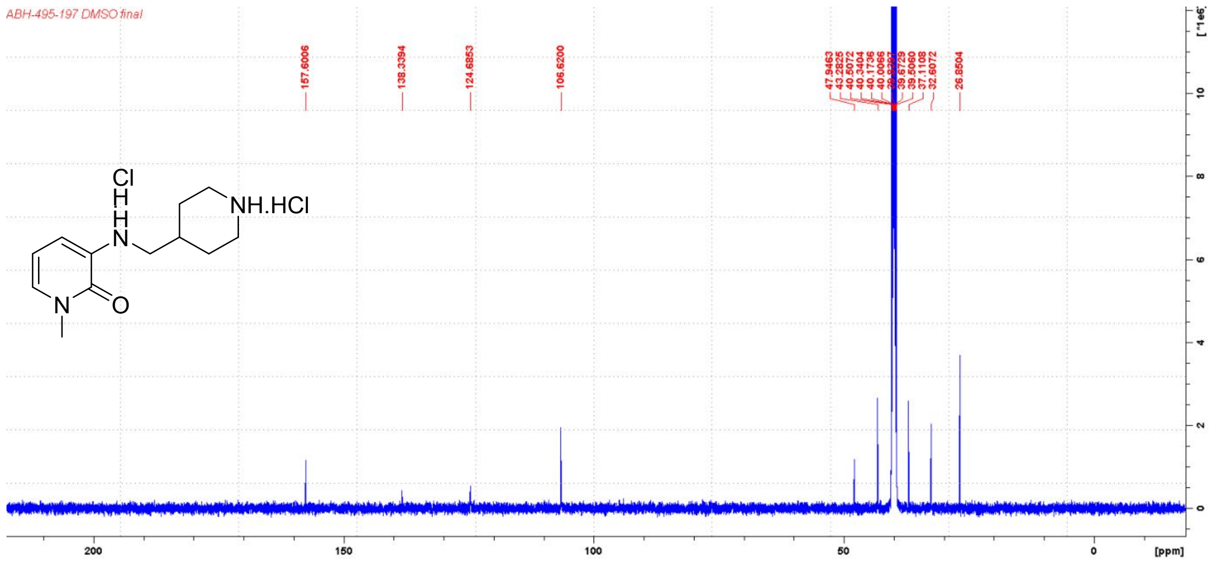

Cmpd 10: ^{1}H NMR ($CDCl_3$)

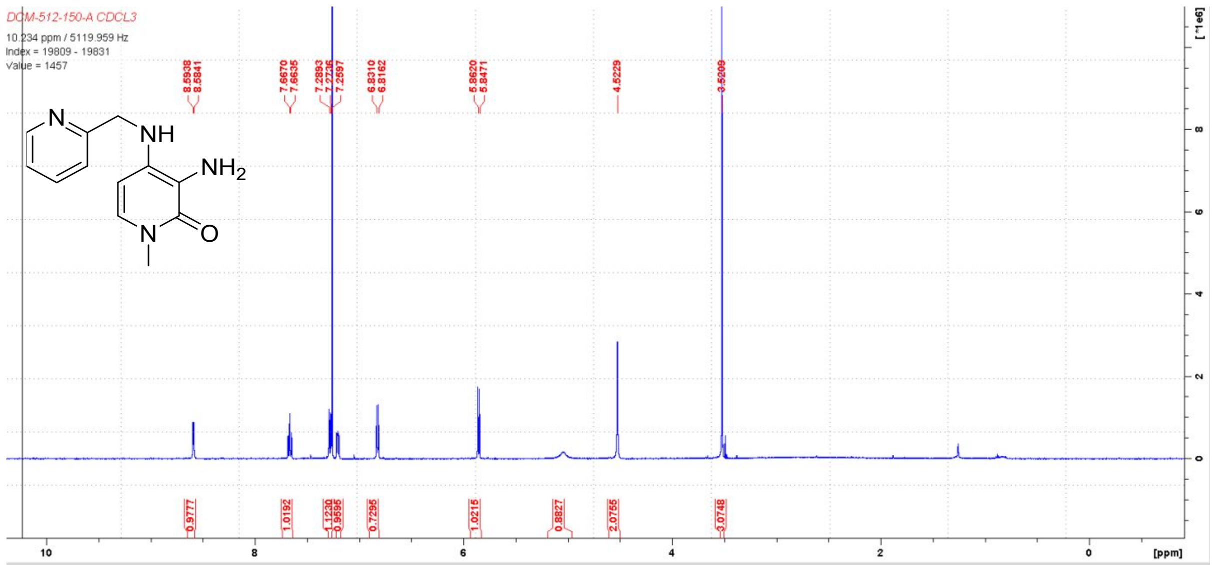

Cmpd 10: ^{13}C NMR ($CDCl_3$)

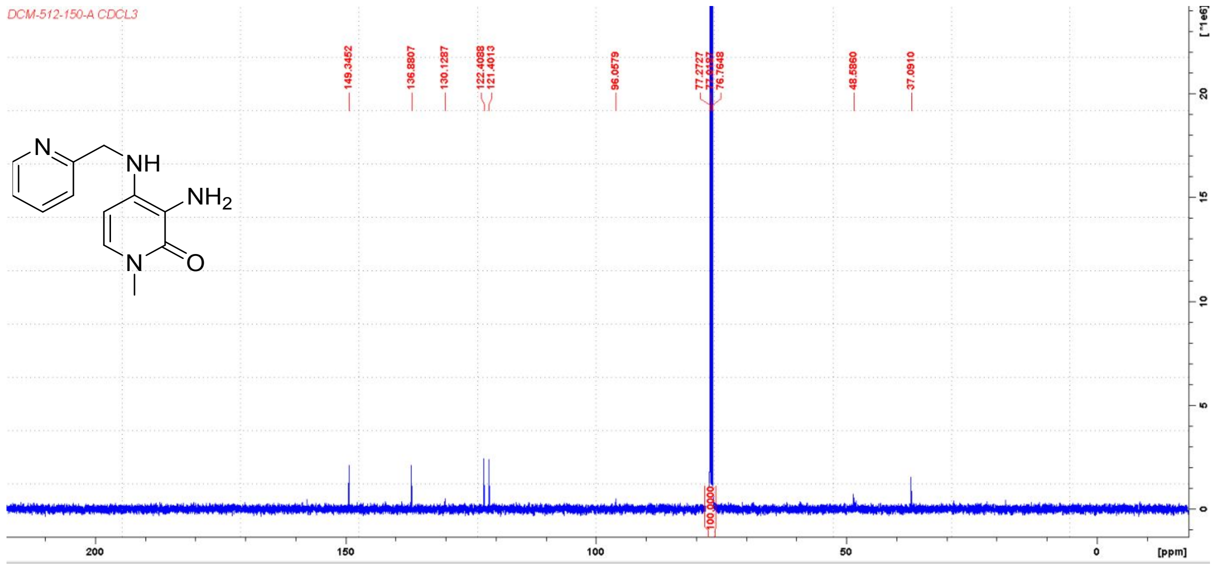

Supplement: Supplementary file 2 — jm2c01357_si_002.pdf [file jm2c01357_si_002.pdf]
